# An Overview of Extracellular Matrix-Based Bioinks for 3D Bioprinting

**DOI:** 10.3389/fbioe.2022.905438

**Published:** 2022-05-11

**Authors:** Haonan Wang, Huaqing Yu, Xia Zhou, Jilong Zhang, Hongrui Zhou, Haitong Hao, Lina Ding, Huiying Li, Yanru Gu, Junchi Ma, Jianfeng Qiu, Depeng Ma

**Affiliations:** ^1^ Department of Radiology, The Second Affiliated Hospital of Shandong First Medical University, Tai’an, China; ^2^ Department of Clinical Medicine, Shandong First Medical University and Shandong Academy of Medical Sciences, Tai’an, China; ^3^ Department of Radiology, Shandong First Medical University and Shandong Academy of Medical Sciences, Tai’an, China

**Keywords:** extracellular matrix, 3D bioprinting, tissue engineering, bioink, biomaterial, tissue regeneration

## Abstract

As a microenvironment where cells reside, the extracellular matrix (ECM) has a complex network structure and appropriate mechanical properties to provide structural and biochemical support for the surrounding cells. In tissue engineering, the ECM and its derivatives can mitigate foreign body responses by presenting ECM molecules at the interface between materials and tissues. With the widespread application of three-dimensional (3D) bioprinting, the use of the ECM and its derivative bioinks for 3D bioprinting to replicate biomimetic and complex tissue structures has become an innovative and successful strategy in medical fields. In this review, we summarize the significance and recent progress of ECM-based biomaterials in 3D bioprinting. Then, we discuss the most relevant applications of ECM-based biomaterials in 3D bioprinting, such as tissue regeneration and cancer research. Furthermore, we present the status of ECM-based biomaterials in current research and discuss future development prospects.

## Introduction

**FIGURE 1:**
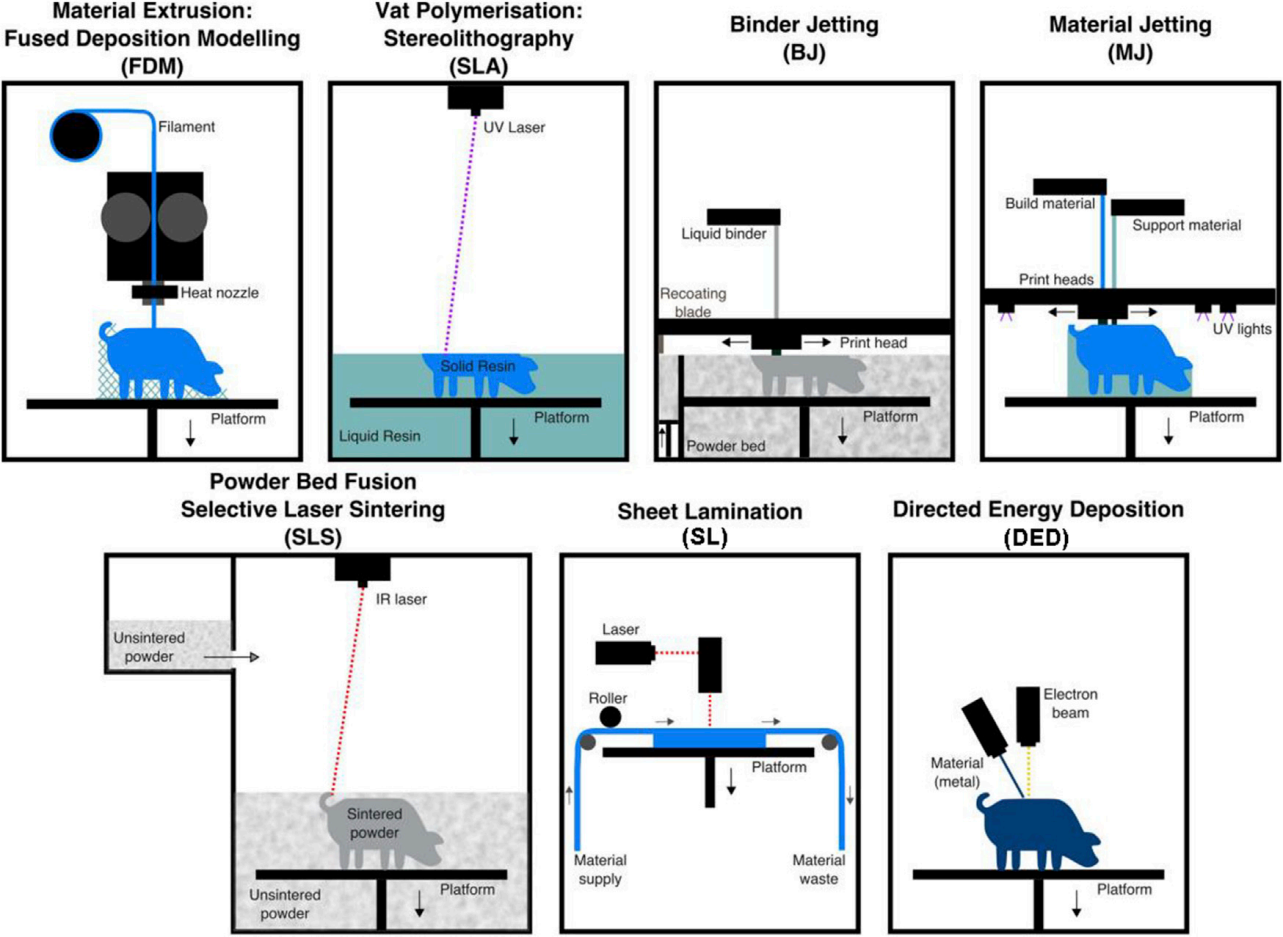
Illustrations of the seven types of 3D printing technologies. Adapted with permission from (Carew and Errickson, 2020).

**FIGURE 2:**
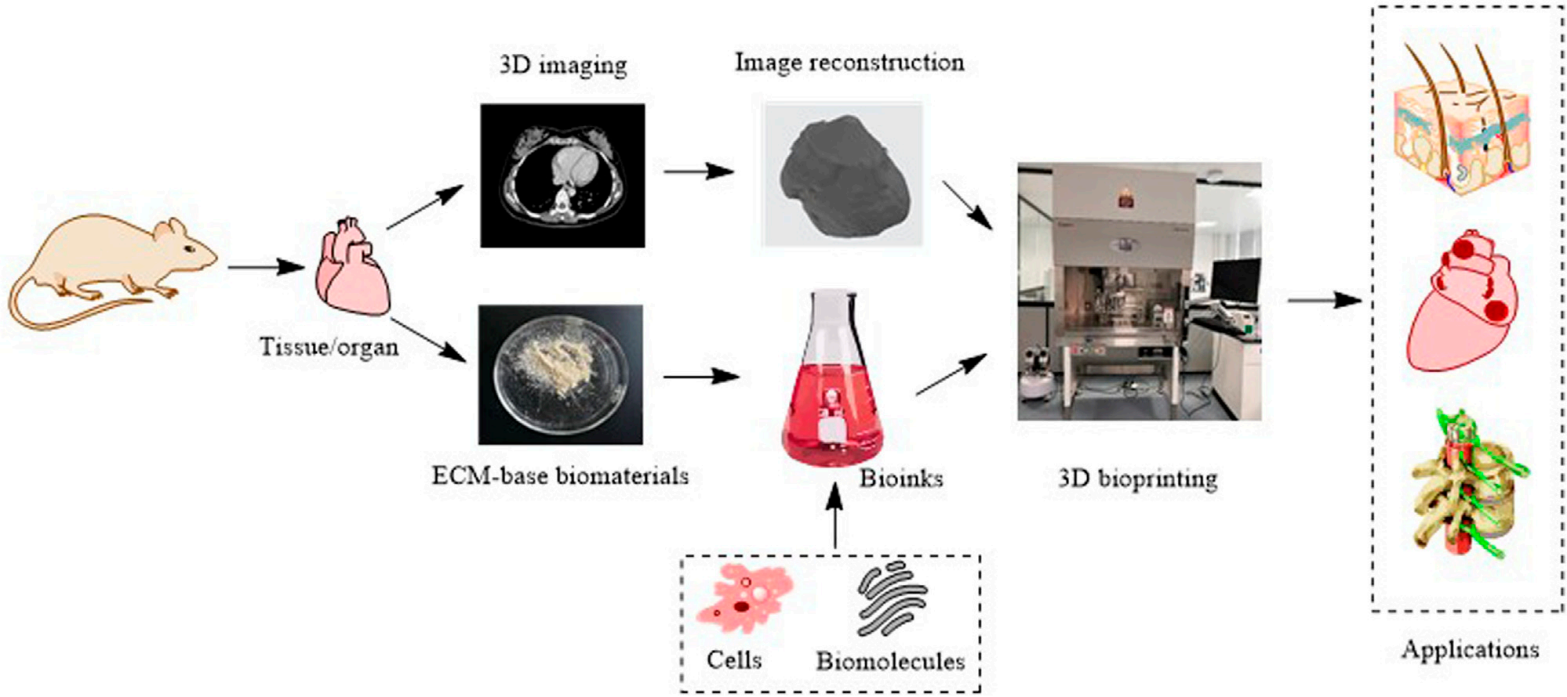
The schematic of 3D bioprinting.

Three-dimensional (3D) printing, also called additive manufacturing, is a rapid prototyping technology that creates 3D physical objects layer by layer (Chatterjee and Ghosh, 2020). Since the invention of the first 3D printing technology, stereolithography, in 1983, numerous 3D printing technologies have been developed (Kulkarni et al., 2000). According to different principles, the American Society for Testing and Materials International Standard has classified 3D printing technologies into seven categories: 1) material extrusion, 2) powder bed fusion, 3) vat photopolymerization, 4) material jetting, 5) binder jetting, 6) sheet lamination, and 7) energy deposition. These technologies are described in Table 1 and illustrated in Figure 1. 3D printing can quickly fabricate various structures with little waste of material. Coupled with the gradual reduction of the cost and personalized customization, 3D printing has become widely used in many fields, especially in medical fields, such as tissue engineering (Ma et al., 2022). Thus, 3D bioprinting came into being. An important branch of 3D printing, 3D bioprinting is a combination of 3D printing and biology (Munaz et al., 2016). It generates biological tissues or organs using bioinks to achieve the purpose of mimicking their natural counterparts in structure and function (Figure 2) (Matai et al., 2020). The natural or synthetic biomaterials loaded with living cells are called bioink, and are the raw material of 3D bioprinting processes (Hospodiuk et al., 2017). Therefore, the selection of bioinks is a basic factor to be considered in 3D bioprinting, and it is also one of the biggest challenges. Such materials must have good printability, biocompatibility, and excellent mechanical and degradation properties (Mao H. et al., 2020).

**TABLE 1:** Summaries of seven types of 3D printing technologies (Hubs, 2018; Additive Manufacturing Research Group, 2019; Carew and Erlrrickson, 2020).

Categories	Typical technologies	Description	Typical materials	Characteristics	
				Advantages	Disadvantages
Material extrusion	Fused Deposition Modeling (FDM)	The material is melted, and deposited *via* a heated nozzle	Thermoplastics	Common material Low cost	Rough surface, Warping
Powder bed fusion	Selective laser sintering (SLS)	The powder of material is fused by a high energy source	Thermoplastics, metal powders, caramic powders	No support Scalable	Higher cost
Vat photopolymerization	Stereolithography (SLA)	Liquid photopolymer material is selectively cured using a light source	Liquid resin	Relatively quick Fine details	Require supports UV sensitive
Material jetting	Material jetting (MJ)	The droplets of liquid photosensitive fusing agent are deposited on a powder bed and cured by light	Liquid photopolymer material	High accuracy Multiple material	High cost Brittle
Binder jetting	Binder jetting (BJ)	The liquid binding agent is deposited on a bed of powder material, which is later sintered together	Liquid bonding agent	No support No warping or shrinking	Post processing
Sheet lamination	Laminated object manufacturing (LOM)	The sheets of material are cut to shape and laminated together	Paper, metal, plastic	Multi-material layers Fast	Limited materials
Direct energy deposition	Direct energy deposition (DED)	The material is fused simultaneously deposited	Polymer, ceramic, metal	Range of materials Larger parts	High cost Poor surface

At present, the materials used for 3D bioprinting are generally divided into two categories: naturally derived biomaterials (such as collagen, hyaluronic acid, and gelatin) and synthetic biomaterials (such as polylactic acid, polycaprolactone, and polyurethane). These materials have different advantages and disadvantages. The naturally derived biomaterials have good biocompatibility, but their mechanical properties are weak. Despite the poor biocompatibility of synthetic biomaterials, they have adjustable properties (Park et al., 2022). To successfully construct a functional living tissue microenvironment, it is necessary to simulate the composition and distribution of target tissues or organs. No single material can replace all the functions of native tissues and organs.

Biological tissues are composed of various cell types and the extracellular matrix (ECM). The ECM is a 3D network consisting of extracellular macromolecules and minerals. One of its roles is to provide structural and biochemical support to surrounding cells (Xing et al., 2020). The ECM has several applications in improving wound healing and tissue reconstruction. Especially in tissue regeneration, the biocompatibility, and degradation properties of the ECM and its derivatives are considered superior to those of synthetic materials. Compared to synthetic materials, ECM-based biopolymers can mitigate foreign body responses by presenting the ECM molecules at the interface between the material and tissue, and can also evoke innate immune responses to replace the implanted matrices with new ECMs. Therefore, ECM-based biomaterials are of great significance in 3D bioprinting (Xing et al., 2020).

In this review, we first discuss ECM-based 3D bioprinting materials, including natural ECM, ECM derivatives, and ECM composites and blends. Then, some applications of ECM-based biomaterials are summarized along with the most recent research works. Finally, the challenges and future prospects for ECM-based 3D bioprinting materials are discussed.

### Extracellular Matrix-Based 3D Bioprinting Materials

The ECM is an intricate network composed of an array of multidomain macromolecules organized in a cell/tissue-specific manner. The structures and properties of the ECM vary from cell to cell (Yue, 2014). For example, the ECM of the cornea is a transparent and soft sheet, and the ECM of the bone is hard like a rock. Therefore, the research and development of 3D printing materials with native ECM components, structures, and biological properties are very important.

### Native Extracellular Matrix and Derived Biomaterials

As the natural microenvironment in which cells reside, the ECM of each tissue and organ is unique in both physicochemical and biological properties (Watt and Huck, 2013). The ECM provides cells with mechanical support and biochemical signals to promote cell proliferation and differentiation, among other functions (Gattazzo et al., 2014). The natural ECM obtained from human skin, heart, lung, and other organisms through mechanical disruption, enzymatic digestion, chromatography, and precipitation has been studied by researchers in the context of 3D bioprinting. For example, Kupfer et al. (2020) used an ECM-based bioink containing collagen methacrylate, laminin-111, and fibronectin to print human-induced pluripotent stem cell–laden structures with two chambers and a vessel inlet and outlet. After the stem cells proliferated to sufficient density, they demonstrated the function of the resulting human luminal muscle pump. This is important for studying cardiac function and remodeling in health and disease.

**FIGURE 3:**
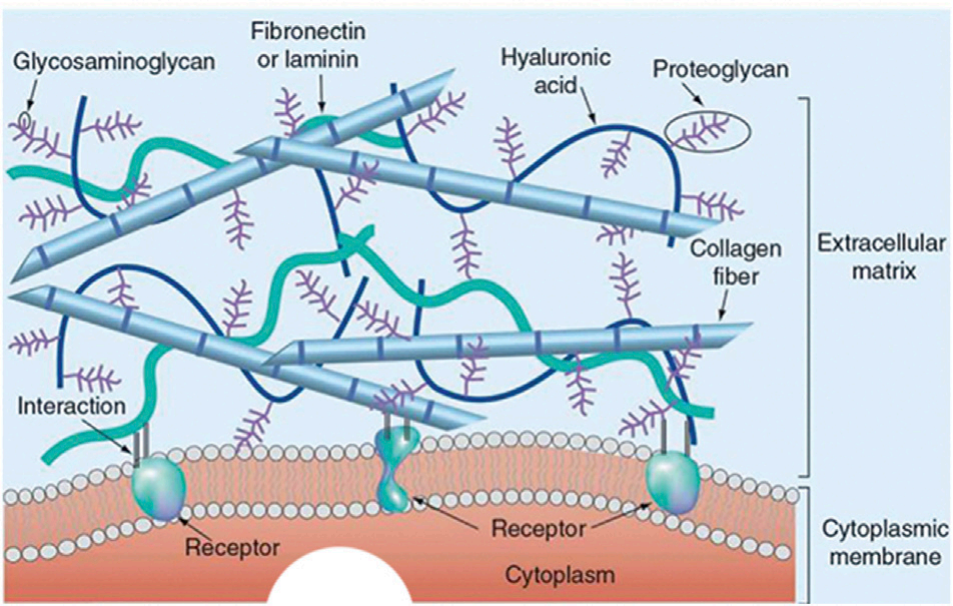
The 3D structure model of the natural ECM. Reprinted with permission from (Aghmiuni and Khiavi, 2017).

**FIGURE 4:**
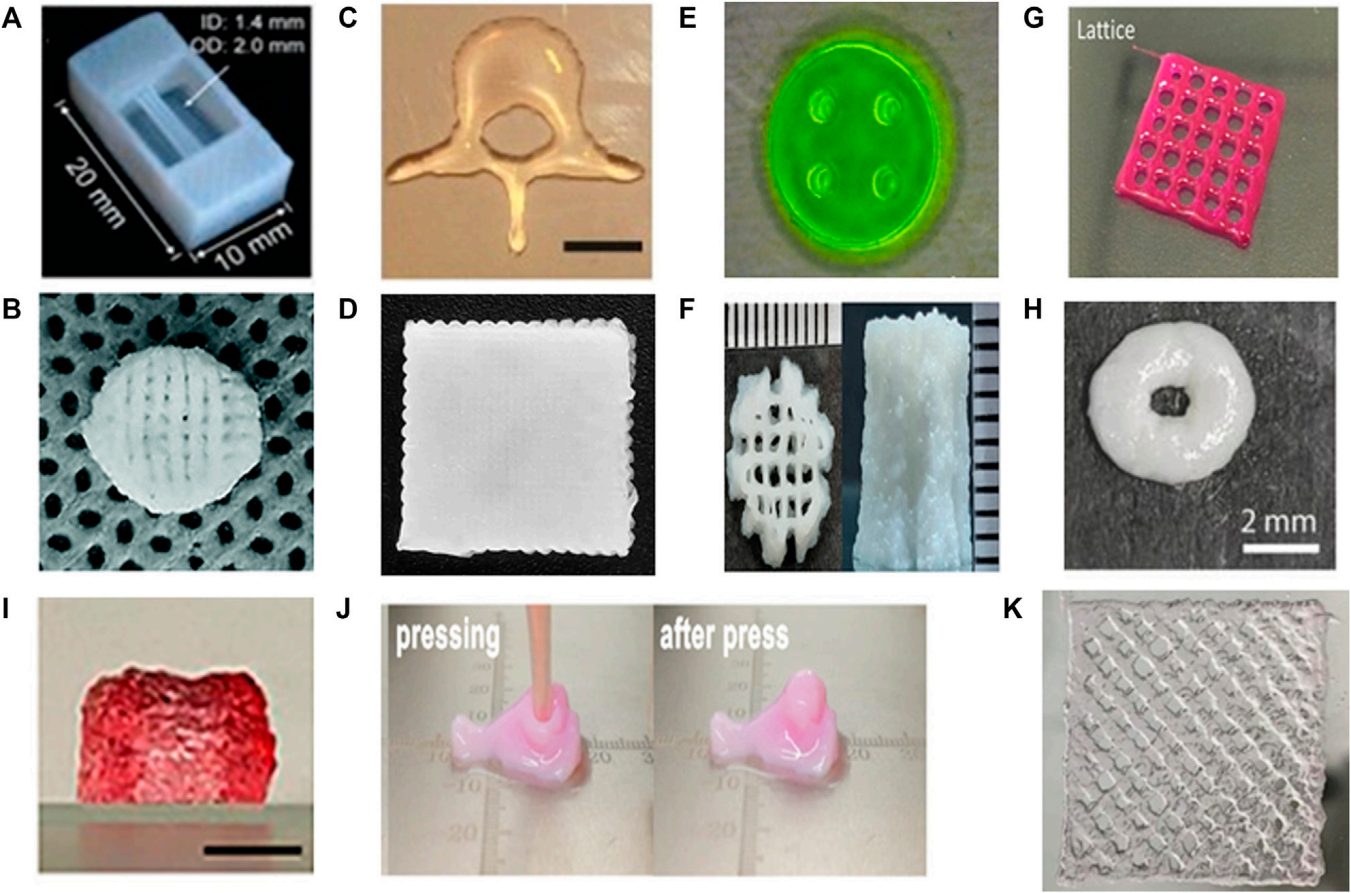
Different ECM-based biomaterial types and resulted constructs. **(A)** Tube construct printed with collagen. Adapted with permission from (Lee et al., 2019). **(B)** Scaffold printed with collagen/heparin sulfate. Adapted with permission from (Jiang et al., 2020). **(C)** The non-porous human L3 vertebrae printed with MeHA. Adapted with permission from (Poldervaart et al., 2017). **(D)** The scaffolds printed with gelatin-alginate-hyaluronic acid. Adapted with permission from (Bertuola et al., 2021). **(E)** The nerve guidance conduits printed with GelMA. Adapted with permission from (Ye et al., 2020). **(F)** The scaffold printed with gelMA and hydroxyapatite (Das and Basu, 2022). **(G)** The scaffold printed with skin-derived dECM bioink. Adapted with permission from (Kim et al., 2018). **(H)** The scaffold printed with liver-derived dECM/PCL bioink. Adapted with permission from (Elomaa et al., 2020). **(I)** The dual cross-linked constructs printed with oxidized hyaluronate (OHA)/glycol chitosan (GC)/adipic acid dihydrazide (ADH)/hyaluronate-alginate hybrid (HAH). The gel constructs maintained their original dimension after 3 weeks at 37°. Adapted with permission from (Kim et al., 2022). **(J)** Nose-shaped construct printed with PU-gelatin. Adapted with permission from (Hsieh and Hsu, 2019). Copyright (2019) American Chemical Society. **(K)** The scaffold printed with tetrameric peptides as bioinks. Adapted with permission from (Rauf et al., 2021).

The native ECM is complex in terms of its composition, and it mainly contains collagen, non-collagen glycoproteins (such as fibronectin, laminin, and tenascin), glycosaminoglycans (such as hyaluronic acid), and proteoaminoglycans (such as asperlecan and aggrecan) (Figure 3) (Choudhury et al., 2018; Dzobo et al., 2019). In 3D bioprinting, bioinks containing isolated ECM components have been widely used (Figure 4). Collagen is the most abundant and ubiquitous protein in the body, accounting for approximately 25–30% of the total vertebrate protein (Ricard-Blum, 2011). Type I collagen is the dominant fibrillar component of the ECM in mammals. It provides cells with a 3D environment that supports cell growth and influences morphology and function. The triple helix structure of collagen provides thermal stability and mechanical strength for cellular functions (Shoulders and Raines, 2009). To manufacture collagen, collagen rich tissues as skin and tendon of mammals are intensively processed by physical and chemical means (Meyer, 2019). Nocera et al. (2018) used collagen isolated from the bovine Achilles tendon to construct scaffolds by 3D printing. A porous mesh of fibrillar collagen was observed using scanning electron microscopy. In addition, the 3D-printed collagen scaffold was not cytotoxic, with cell viability higher than 70% using Vero and NIH 3T3 cells. *In vitro* evaluation demonstrated that the collagen scaffolds had the ability to support cell attachment and proliferation.

Although type I collagen has been widely used in 3D bioprinting, it has limitations, such as poor mechanical properties, low viscosity, and long gelation time (Sorushanova et al., 2019). In recent years, researchers have used different strategies to improve the printing properties of collagen, including chemical modification of collagen, adjustment of pH, temperature, collagen concentration, and mixing of collagen with other materials (Kim et al., 2016). Collagen methacrylamide is a chemically modified collagen with photosensitive groups, which can be rapidly cured by UV irradiation (Drzewiecki et al., 2017). Isaacson et al. (2018) 3D-bioprinted corneal structures from a methacrylated collagen bioink containing encapsulated corneal keratinocytes, which exhibited high cell viability both at day 1 (>90%) and at day 7 (83%) after printing. Diamantides et al. (2017) investigated the effect of pH on the rheological properties of type I collagen bioink. The results showed that the gelation kinetics and final gel moduli of the bioink were highly pH-dependent. It was also found that cell viability in collagen bioink was not affected by the pH. To fabricate multilayered, heterogeneous constructs with high-resolution microchannels (150 µm-1 mm) precisely spaced (500–600 µm) to simulate integrated vascular networks, Attalla et al. (2018) used silicon carbide (SiC) nanoparticles as an adhesive to achieve adhesion between hybrid hydrogel films composed of alginate and collagen by 3D printing. The bonding strength between the mixed hydrogels reached 0.39 ± 0.03 kPa after the introduction of SiC. The hollow microchannels of the hydrogels were not blocked by the SiC nanoparticles, and high cell viability (90.61 ± 3.28%) was maintained in the scaffold.

Hyaluronic acid (HA), another main component of the ECM, is a high molecular weight natural polysaccharide composed of repeating disaccharide units of D-glucuronic acid and N-acetyl-D-glucosamine. Hyaluronic acid has been widely used in tissue engineering because of its biodegradability, biocompatibility, hydrophilicity, and non-immunogenicity. Therefore, HA has been approved by the FDA for other medical applications. For example, HA has been incorporated into many scaffold systems. HA-based scaffolds are biocompatible and can simultaneously perform different biological functions (Unterman et al., 2012). Park et al. (2014) investigated the behavior of chondrocytes to HA hydrogels. They demonstrated that chondrocytes on HA hydrogels exhibited better proliferation and cellular function than cells on non-native ECM hydrogels. In addition, 3D cartilage tissue-mimicking structures consisting of chondrocyte-encapsulated HA hydrogels were bioprinted, and it was found that the viability and function of chondrocytes were well-maintained in the 3D structures up to 14 days *in vitro* (Ozbolat and Hospodiuk, 2016).

However, the precursor solutions of HA hydrogels usually have low viscosity and slow gelation kinetics, which lead to flow before gelation (Kesti et al., 2015). Moreover, HA hydrogels have poor mechanical properties and rapid degradation (Jeon et al., 2007). Therefore, many other approaches have been studied to improve these problems. Poldervaart et al. (2017) modified HA to obtain methacrylate hyaluronic acid (MeHA) with photo-crosslinkable property. Under the irradiation of UV light, the storage modulus and elastic modulus of the gel increased. Subsequently, human bone marrow mesenchymal stromal cells (MSCs) were incorporated into the MeHA hydrogel, and the cell viability was 64.4% after 21 days of culture. Si et al. (2019) synthesized methacrylic anhydride HA (HA-MA) and sulfhydryl-containing HA (HA-SH). A 3D-bioprinted, double-crosslinked, and HA-based hydrogel for wound dressing was prepared by mixing HA-MA and HA-SH at different weight ratios. The test showed that the storage modulus of the HA-SH/HA-MA hydrogel increased with the increase in the HA-MA content. The hydrogel had a high swelling ratio and a highly controllable degradation rate. And the HA-SH/HA-MA hydrogel had promise in wound repair applications.

UV light can cause cell damage, which can affect cell viability. To avoid the use of UV radiation, Petta et al. (2018) prepared a tyramine-modified HA biofunctional ink. The bioink did not require premixing of components or addition of stabilizers. It was first enzymatically crosslinked to tune extrusion properties, followed by visible light-induced crosslinking to achieve final shape fixation. Optimizing printing parameters resulted in 3D constructs with high resolution and shape fidelity that could be seeded with HMSCs. Nedunchezian et al. (2021) also created an HA-based double crosslinking reaction to avoid the use of UV light. First, HA-biotin (HAB) was synthesized via a reaction of HA and adipic acid dihydrazide. Then, HAB and streptavidin were mixed to form a partially crosslinked HA-biotin-streptavidin (HBS) hydrogel. The HBS hydrogels were mixed with sodium alginate and subsequently printed using a bioprinter to form HBSA (HBS + alginate) hydrogel 3D scaffolds. Finally, 3D scaffolds of HBSA hydrogels were submerged into CaCl_2_ solution to obtain a stable 3D HBSAC (HBSA + Ca^2+^) hydrogel scaffolds by ion-transfer crosslinking.

A commonly used 3D bioprinting material, gelatin is a natural protein derived from collagen hydrolysis. Gelatin is non-cytotoxic, water-soluble, and biocompatible, and it promotes cell adhesion with biodegradable properties and low immunogenicity. Moreover, gelatin can replace collagen in 3D bioprinting due to better physical properties. Shin and kang (2018) prepared a series of gelatin-based bioinks for 3D bioprinting and evaluated them in terms of their printability. The results showed that this class of bioinks can produce a line width of about 200 μm and can precisely locate multiple types of cells in 3D structures. These findings suggested that gelatin-based bioink is well-suited for 3D bioprinting.

Gelatin methacrylate (GelMA) has been synthesized by addition of methacrylate groups to the amine t groups of gelatins. GelMA undergoes photoinitiated radical polymerization to form covalently crosslinked hydrogels. GelMA hydrogels are very similar to native ECM, including with respect to cell attachment sites, proteolytic degradability, and presence of matrix metalloproteinase-responsive peptide motifs, which can be used to design tissue analogs (Aljohani et al., 2018). Billiet et al. (2014) studied the 3D bioprinting parameters of GelMA in detail. The results showed that printing pressure and needle shape affected overall cell viability. By tuning these parameters, mechanically stable cell-laden gelatin methacrylamide scaffolds with high cell viability (>97%) could be printed. Liu et al. (2017) reported a novel strategy to directly bioprint cell-laden GelMA constructs with high structural fidelity and enhanced bioactivity using bioinks of GelMA physical gels (GPGs) achieved through a simple cooling process. The GPG bioinks retained their structures and formed very soft constructs at relatively low concentrations (down to 3%) of GelMA.

Gelatins with other modified groups have also been reported. For example, Yan et al. (2018) synthesized a thiolated-gelatin supplemented with peptides amphiphiles to prepare 3D bioprinting bioinks. The bioink could be printed at 4°C and stabilized to last more than 1 month in culture at 37°C.

### Decellularized Extracellular Matrix

Decellularized extracellular matrix (dECM) is produced by removing all cellular components from the tissue or organ while preserving the composition and integrity of the native ECM. There are three main decellularization methods: physical, chemical, and enzymatic approaches as well as combinations of them. The chemical methods of decellularization can largely be divided into three categories where tissue samples can be treated with surfactant or acid or base reagents, all of which can effectively remove cells and genetic material (Gilpin and Yang, 2017). Physical method such as freeze-thaw and osmotic pressure can result in cell lysis without significantly disrupting the ultrastructure of the tissue (Kim et al., 2019). Enzymatic decellularization is used to degrade cell and remove cellular debris and remnants from the ECM. Nucleases and proteases are the most widely used enzymes for enzymatic decellularization (Kim et al., 2019). The methods have different material requirements, advantages, and disadvantages (Abaci and Guvendiren, 2020).

Although ECM-mimicking biomaterials have demonstrated good biocompatibility and printability, it is still extremely difficult to accurately reproduce the composition and structure of the native ECM in the complex system of the human body. dECMs contain a variety of growth and differentiation agents that modulate cell function, and they have the potential to meet all clinical performance requirements. Currently, dECMs derived from many different organs, including the skin, bone, heart, liver, and blood vessel, have been used for 3D bioprinting. Pati et al. (2014) prepared three specific dECM bioinks from adipose, cartilage, and cardiac tissue and developed a method for the bioprinting of cell-laden constructs with dECM bioinks. The results demonstrated that this method is capable of providing an optimized microenvironment conducive to the growth of 3D structured tissues and helping recreate intrinsic cellular morphology and function. Won et al. (2019) developed a dECM bioink derived from porcine dermis tissue, and mixed with human dermal fibroblasts (HDFs) for 3D bioprinting. Survival and proliferation of HDFs in the 3D construct were investigated. The cells showed over 90% viability and proliferation, and gene expression related to skin morphology and development had been enhanced. These results showed the positive effects of the bioink on skin morphology and development.

Although many researchers have demonstrated 3D bioprinting of dECM from different organs with promising results, dECM-based tissue or organ bioprinting has not been well established. The main problem is the weak mechanical properties of physically crosslinked dECM. One of the methods to solve the above problem is using a framework printed with high mechanical strength biomaterials (e.g., PCL, silicone rubber) to maintain the structure of the dECM. Pati et al. (2014) printed a PCL framework. Then, the cell-laden dECM precursor solution was deposited on the framework to fabricate cartilage tissue structures. To reconstruct functional small-diameter blood vessel substitutes, Xu et al. (2018) used silicone ink to bioprint a support scaffold with a double-layer circular structure. Human aortic vascular smooth muscle cells, human umbilical vein endothelial cells (HUVECs), and human dermal fibroblasts-neonatal were separately used to form the media, intima, and adventitia of blood vessels through perfusion into the corresponding location of the supporting scaffold. In addition, the dECM bioink was printed into a Pluronic F-127 support frame to build a thick structural model with multi-level vascular channels. After the removal of Pluronic F-127 as a sacrificial material, thick tissue constructs with multilevel hollow channels were obtained.

Another effective strategy to improve the mechanical strength of dECM-based bioinks is to combine the dECM with other synthetic polymers or active molecules (Kim H. et al., 2021). Yu et al. (2019) developed a photo-crosslinkable tissue-specific dECM bioink using a digital light processing-based 3D printing method. The ink consisted of photo-crosslinkable GelMA, dECM, and photoinitiator lithium phenyl-2,4,6 trimethylbenzoylphosphinate. Combining tissue-matched dECM bioinks with human-induced pluripotent stem cell derived cells enables the design of physiologically relevant functional human tissues for applications in biology, regenerative medicine, and diagnostics.

Shin et al. (2021) reported a hydrogel bioink containing porcine cardiac acellular extracellular matrix (cdECM), Laponite-XLG nanoclay, and poly (ethylene glycol)-diacrylate (PEG-DA) components. Among them, Laponite-XLG nanoclay had shape fidelity and high resolution of the constructs, which was achieved by increasing the shear storage modulus and viscosity of the cdECM-based hydrogels. PEG-DA further enhanced the modulus of the hydrogel by photopolymerization after printing. The results showed that the encapsulated human cardiac fibroblasts survived both extrusion and photopolymerization to show >97% viability after 7 days, demonstrating the cytocompatibility of the cdECM composite bioinks. This was also a way to improve the mechanical strength of the dECM.

Bioprinting using dECM is an attractive option that opens up new avenues for tissue reconstruction. However, there is a lack of reproducibility and standardization in the decellularization process; lack of control over printability of dECM-based bioinks and the mechanical stability of the printed dECM structures; and difficulty of large-scale production due to tissue specificity of dECM from different tissue (Han et al., 2019). Therefore, widespread use of dECM-based bioprinting is currently limited. Further research is required to make dECM a viable product for 3D bioprinting.

### Extracellular Matrix-Based Multicomponent Biomaterials

Human tissue has a complex structure, which creates challenges with respect to the properties of the bioprinted materials. A single biomaterial in bioinks cannot usually meet all mechanical and functional requirements, which are essential to produce biomimetic tissue-like constructs (Ashammakhi et al., 2019). One of the most important strategies to solve these problems is to use multimaterial bioinks. ECM-based blends/composite bioinks with high performance have been generated by blending other materials with unique properties and by the inclusion of fillers and additives with distinct properties.

Natural biological materials (such as chitosan, alginate, and agarose) have the advantages of biological activity, degradation, and non-toxic degradation products. They have high similarity and excellent biocompatibility with the ECM. Therefore, hybrid inks containing ECM-based materials and natural biomaterials are widely used in 3D bioprinting. Köpf et al. (2016) combined agarose and type I collagen in order to prepare a 3D-printed hydrogel mixture. The results showed that the mixture containing 0.5% agarose and 0.2% collagen type I displayed sufficient cell spreading and printing accuracy. Kreimendahl et al. (2017) presented tailored bioinks that could be printed in 3D and exhibit cell-induced vascularization capability. The bioinks contained agarose, type I collagen, human umbilical vein endothelial cells, and human dermal fibroblasts. The results showed that extensive capillary network formation was observed in hydrogel blends. The storage moduli of the bioink were significantly increased compared to those of the corresponding single components.

Alginate is a natural, seaweed-derived, and ion-sensitive anionic polysaccharide (Neufurth et al., 2014). Alginate can transiently form a hard gel with CaCl_2_ *via* sodium–calcium ion exchange reaction at ambient temperature (Demirtaş et al., 2017). Therefore, alginate can provide a cytoprotective effect against processing pressure stress. Kalkandelen et al. (2019) investigated gelatine/sodium alginate hydrogels reinforced with β-Tricalcium Phosphate to form 3D bone tissue. *In vitro* bioassays with a human osteosarcoma cell line, SAOS-2, were performed to determine the biocompatibility of the constructs. It was found that cell viability rates for all constructs were increased. A 3D-printed hydrogel with self-healing ability was prepared by Roh et al. (2021). The hydogel was composed of oxidized hyaluronate (OHA), glycol chitosan (GC), and adipic acid dihydrazide. The addition of alginate (ALG) to this self-healing hydrogel was useful for the dual crosslinking system, which enhanced the structural stability of the gels without the loss of their self-healing capability. In addition, hyaluronate-alginate hybrid (HAH) polymers were used to replace the ALG mentioned above, and it was found that the storage shear modulus of the OHA/GC/ADH/HAH hydrogels was significantly improved in addition to maintaining the self-healing ability. *In vitro* chondrogenic differentiation of ATDC5 cells encapsulated in 3D-printed constructs was dependent on the molecular weight and concentration of the HAH in gels (Kim et al., 2022).

Apart from the aforementioned materials, other natural biomaterials such as silk fibroin (Sun et al., 2018), cellulose (Fermani et al., 2021), and gellan gum (Robinson et al., 2020) could also be mixed with ECM-based materials to prepare bioinks.

Synthetic biomaterials are less restrictive in 3D printing because their structure and properties can be adjusted according to needs. Thermoplastic polymers, such as PLA, PCL, and PU, are the most commonly used materials in 3D bioprinting. Synthetic biomaterials are printed *via* fused deposition modeling or regular extrusion. In bioprinting, scaffolds printed from synthetic materials often act as a mold surrounding the bioink to prevent it from spilling or as a rigid individual layer to separate the two bioink layers (Shim et al., 2016; Cunniffe et al., 2017).

PCL is a nontoxic polymer with remarkable stability, and it is also fairly inexpensive. 3D-printed PCL scaffolds have a comparable compactness to bone that result in bone regeneration and cell ingrowth capabilities (Guvendiren et al., 2016). Therefore, for cartilage injury that is difficult to self-repair, Cao et al. (2021) proposed a biphasic scaffold consisting of PCL/GelMA to support cartilage regeneration using a co-culture of bone marrow mesenchymal stem cells and costal chondrocytes. The PCL/GelMA scaffold showed excellent cartilage regeneration ability and made Young’s modulus comparable to that of native cartilage. However, in aqueous 3D printing, the tendency of GelMA to physical gelation makes it necessary to keep it at a low concentration in use, thus reducing 3D printing resolution (Zhao et al., 2016). Elomaa et al. (2020) developed GelMA/PCL-MA hybrid resins and used them to print cell-free tissue scaffolds that mimic the structure of physiological small intestinal villi. The results showed that the presence of PCL-MA in the hybrid resin improves the 3D printing fidelity compared to neat GelMA resins, and GelMA provided the hybrid materials with enhanced swelling and proliferation of seeded cells. Wiggenhauser et al. (2019) added the dECM of porcine nasal cartilage to 3D-printed PCL scaffolds. The scaffolds were seeded with human primary nasoseptal chondrocytes. The results showed that cells attached and proliferated on the scaffolds, and evidence of cartilage tissue formation on the PCL/dECM scaffolds was found. This provides a method for cartilage regeneration in facial reconstruction surgery.

Polyurethane (PU) is a 3D-printable biodegradable elastomer with thermosetting properties and excellent biocompatibility and mechanical properties. Chen et al. (2021) employed decellularized cartilage extracellular matrix and waterborne polyurethane (WPU) to construct WPU-ECM scaffolds by 3D printing. It was found that WPU-ECM scaffolds with a hierarchical macroporous structure could recreate a favorable microenvironment for cell adhesion, proliferation, differentiation, and ECM production. *In vivo* studies further demonstrated that the WPU-ECM scaffold successfully regenerated hyaline cartilage in a rabbit model. In addition, Yun et al. (2021) prepared 3D-printed PLA scaffolds and investigated the role of PLA scaffolds with and without HA in a rabbit calvarial model. The results showed that the new bone area of the rabbits transplanted with PLA/HA scaffolds were significantly higher than that of the control group. The printed PLA scaffold was biocompatible and integrated well with the bone defect margin.

Many studies have also been conducted using other synthetic biomaterials (such as ABS, PEEK, and PEG) in combination with ECM-based materials so that the resulting composites possess the desired physical and chemical properties that can contribute significantly to 3D bioprinting (Li et al., 2018; Piluso et al., 2021).

Some nanomaterials have also been doped into ECM-based biomaterials to tune the performance of 3D bioprinted structures. Hydroxyapatite has been widely used in bone 3D printing as the main component of natural bone tissue. Huang et al. (2021) reported the development of gelatin/hydroxyapatite (HAP) hybrid materials by microextrusion 3D bioprinting and enzymatic crosslinking as the scaffold for human umbilical cord blood-derived mesenchymal stem cells (hUCB-MSCs). The scaffold supports the adhesion, growth, and proliferation of hUCB-MSCs and induces their chondrogenic differentiation *in vitro*. This conclusion was confirmed by the study of Cakmak et al. (2020). They used PCL, gelatin, bacterial cellulose (BC), and different hydroxyapatite concentrations to fabricate a novel PCL/GEL/BC/HA composite scaffold using 3D printing technology. 3D scaffolds with an ideal pore size (∼300 µm) for use in bone tissue engineering were generated. Jae-Woo Kim et al. (2021) prepared biomimetic composite scaffolds *via* 3D printing of gelatin/hyaluronic acid/hydroxyapatite. The microstructures of the scaffolds showed an ECM-mimetic structure with a wrinkled internal surface and a porous hierarchical architecture. The composite scaffolds could be used as new bone scaffolds in bone regeneration.

Carbon-based nanomaterials have been frequently incorporated into bioinks due to their excellent electrical and mechanical properties (Blyweert et al., 2021). In addition to their ability to promote cartilage differentiation, they have numerous applications in nerve and muscle tissue engineering (Lu et al., 2021). Uz et al. (2019) fabricated gelatin and graphene-based nerve regeneration conduits/scaffolds possessing 3D microstructures and mechanical properties using 3D printing. The results suggested that electrical stimuli applied within the 3D gelatin matrix enables enhanced differentiation and paracrine activity, leading to promising nerve regeneration strategies. Li et al. (2020) reported the use of carbon nanotubes to construct biomimetic blood vessels. The hybrid bioink prepared with gelatin, sodium alginate, and carbon nanotubes was manufactured into cylindrical scaffolds through 3D printing. Mouse epidermal fibroblasts were inoculated into the hollow tubular scaffolds to fabricate engineered blood vessels. The results demonstrated that the proper doping of carbon nanotubes could effectively improve the mechanical properties of the composite scaffolds. A small amount of doped carbon nanotubes had little effect on cytotoxicity.

Silica is also an important additive in 3D bioprinting to tune the properties of biomaterials. Banche-Niclot et al. (2021) combined PEG-coated silica into type I collagen to obtain bioinks for 3D-printed bone scaffolds. Nelson et al. (2021) use silica-gelatin hybrid ink to produced 3D grid-like scaffolds using a coupling agent, (3-glycidyloxypropyl)trimethoxysilane, to form covalent bonds between the silicate and gelatin co-networks, which improved the mechanical properties of the scaffold. In addition, tricalcium phosphate (Kalkandelen et al., 2019), metal nanoparticles (Zhu et al., 2017), bioceramic materials (Diloksumpan et al., 2020), and others have been added to biomaterials to improve the mechanical, chemical, and electrical properties of inks.

### Synthetic Peptide Biomaterials

The various combinations of amino acids allow the design and synthesis of a large number of peptides with different biological functions. These peptides with hydrophilic or hydrophobic amino acid sequences can self-assemble to form functional materials, which are widely used in biomedical fields including antibacterial, wound healing, drug delivery, and bioimaging (Das and Gavel, 2020). Particularly, short peptides consisting of 2-7 amino acids as hydrogels have been used to mimic ECM for 3D bioprinting. Loo et al. (2015) reported a lysine-containing hexapeptide bioink that self-assembled to form stable nanofibrous 3D hydrogels. The biocompatible hexapeptide-based hydrogel scaffold supported the growth and proliferation of human stem cells and ensured cell viability during printing process. Arab et al. (2018) used two tetrapeptide synthetic biomaterials to self-assemble into nanofibrous hydrogels to mimic the natural collagen. The results showed that the hydrogels maintained cell viability and promoted the growth and alignment of mouse myoblasts. However, due to poor mechanical properties and slow gelation process, peptide-based hydrogels still face great challenges in 3D bioprinting (Chivers and Smith, 2019).

In order to improve the weak mechanical strength and poor printability of short peptides, Jian et al. (2019) designed and synthesized two 9-fluorenylmethoxycarbonyl dipeptides with oppositely charged terminal residues to achieve situ gelation by electrostatic interactions between the two dipeptides. The elastic modulus of the hydrogel was tunable from 4 to 62 kPa to simulate the natural environment of various cell types. Ghalayini et al. (2019) prepared self-assembled peptide nanoparticles and incorporated them into peptide hydrogels for 3D printing. The results showed that the peptide nanoparticles were able to withstand the stresses involved in the printing process.

To improve the cell damage caused by high shear forces during the printing of peptide bioinks, Rauf et al. (2021) developed an *in situ* 3D bioprinting technique that utilizes physiological buffers and works at body temperature. By printing two tetrameric peptide bioinks containing human skin fibroblasts, an increased rate of cell proliferation was found. This is due to the better ECM-like environment provided by the tetrameric peptide bioink.

## Application of Extracellular Matrix-Based Bioprinting Materials in 3D Bioprinting

**FIGURE 5:**
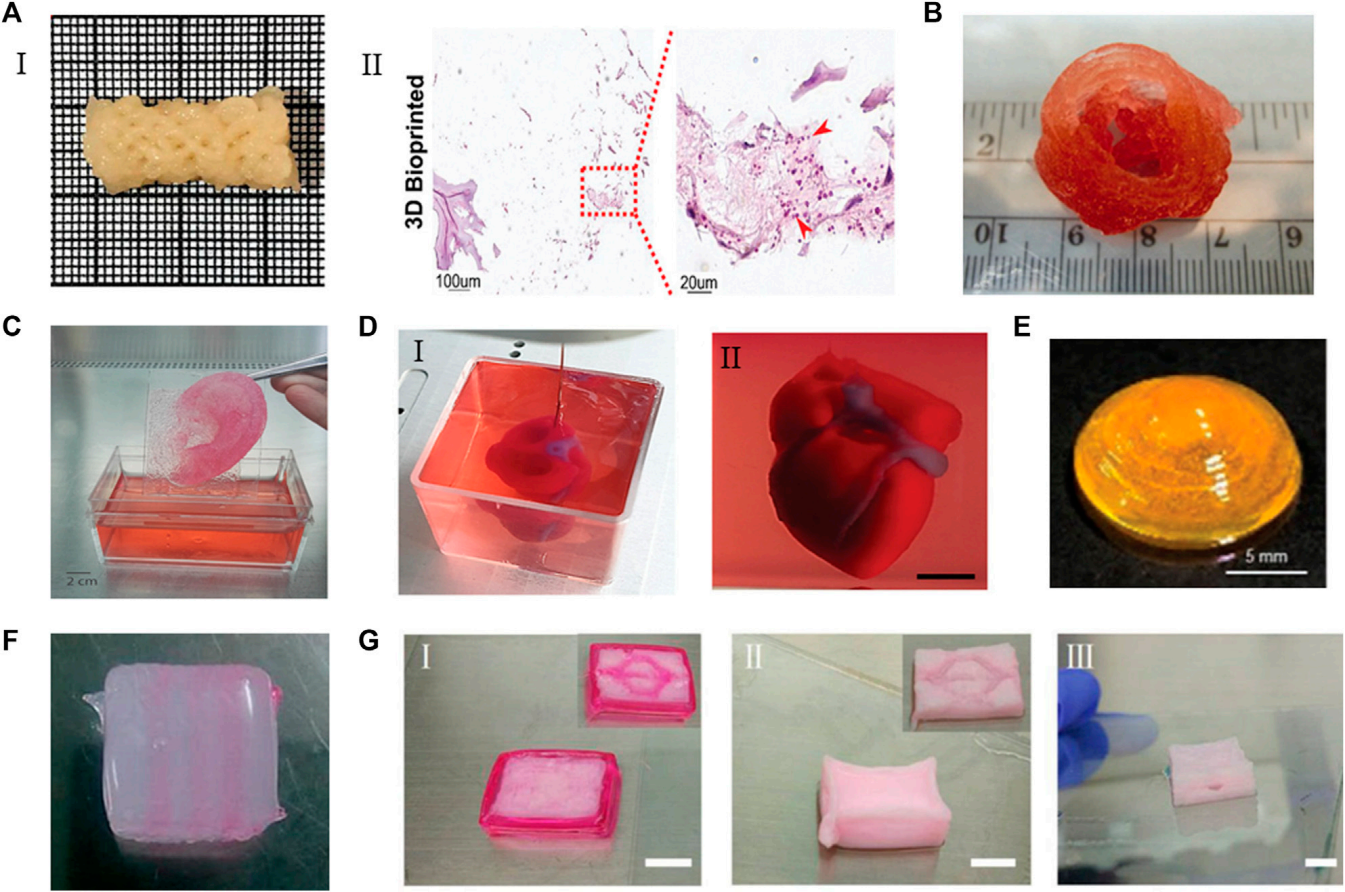
The applications of ECM-based bioinks. **(A)** (I) A clavicle bone scaffold bioprinted with BPs GelMA-based bioink. (II) The scaffold stained for H&Eafter 28 days, the number of cells increased. Adapted with permission from (Ratheesh et al., 2020). **(B)** The aortic valve conduit bioprinted with bioink containing alginate/gelatin hydrogel and aortic root sinus smooth muscle cells and aortic valve leaflet interstitial cells. Adapted with permission from (Duan et al., 2013). **(C)** Adult size ears (8 cm) printed with bioink containing bovine gelatin/alginate/fibrinogen and human fibroblasts. Adapted with permission from (Pourchet et al., 2017). **(D)** A heart printed with bioink containing dECM and iPSCs-derived cardoimyocytes and ECs. Adapted with permission from (Noor et al., 2019). **(E)** The curved cornea based on the eyeball printed with dECM-based bioink. Adapted with permission from (Kim H. et al., 2021). **(F)** The multilevel vascular structures, and **(G)** multibranch vascular channels printed with bioink containing dECM/Pluronic F127 and endothelial cells. Adapted with permission from © 2018 by the (Xu et al., 2018). Licensee MDPI, Basel, Switzerland.

3D bioprinting has shown huge potential in the field of tissue and organ regeneration (Sung et al., 2021). The ECM, as the natural environment in which cells exist, provides cells with structural support and biochemical signals and promotes a series of important cellular processes, such as proliferation, migration, proliferation, and differentiation (Lu et al., 2011). Therefore, ECM-based biomaterials are ideal for tissue and organ regeneration printing materials (Figure 5). In this section, we describe some important applications of bioinks containing different ECM-based materials in the fabrication of tissue structures.

### Bone

Bone tissue is an essential part of the human body and plays a role in mechanical support and protection, mineral homeostasis, and hematopoiesis (Romanazzo et al., 2021). Over the past few years, many efforts have been made to use 3D printing technology to regenerate damaged bone tissue. Traditional 3D printing technologies first prepare the scaffold; then, the cells are infused and inoculated prior to implantation. However, this method cannot ensure uniform distribution of cells on the scaffold, making it difficult to obtain ideal new tissue (Ghorbani et al., 2021). 3D bioprinting technology can print cells and scaffolds at the same time; different cells can be stacked in specific locations; and the biological behavior and performance of cells can be modulated by active agents (Bendtsen et al., 2017). Ratheesh et al. (2020) developed a bone particle (BP)-GelMA-based bioink as a personalized therapeutic strategy for bone regeneration. The 15% BP content enabled reproducible bioprinting at 10% and 12.5% w/v GelMA concentrations and maintained cell viability in BPs throughout the bioprinting process. These cells were also able to migrate from BP, colonize the GelMA hydrogel, and maintain their osteogenic differentiation capacity. As a major component of the ECM, HA plays a key role in maintaining cartilage homeostasis by regulating cellular functions, including promoting the chondrogenic phenotype and the production and retention of matrix components (Knudson, 2003). Antich et al. (2020) developed a novel HA-based bioink for 3D bioprinting of cartilage tissue. To produce cartilage structures with optimal mechanical properties, HA-based bioinks were co-printed with PLA. HA-based bioinks were found to improve cell function by increasing the expression of chondrogenic gene markers and specific matrix deposition and thus tissue formation. In addition, GelMA has been used for 3D-printing of cartilage tissue. For example, an interpenetrating network hydrogel composed of alginate and GelMA reinforced with a PCL fibrous network has a balance that matches or approximates that of native articular cartilage along with possessing dynamic mechanical properties. Co-cultures of bone marrow-derived stromal cells and chondrocytes were added to this hydrogel to form cartilage tissue structures (Schipani et al., 2020).

Bone tissue engineering approaches using mesenchymal stem cells (MSCs) have attracted tremendous interest in recent years, as MSCs can differentiate into osteoblasts *in vivo*, and autologous MSCs can be isolated without severe donor site morbidity. In addition, animal studies have shown that MSTCs support bone healing in critical-sized bone defects (Wakitani et al., 1994). Zhang X. et al (2021) prepared bioinks containing silk fibroin and dECM (SF-dECM bioinks) mixed with bone marrow MSCs (BMSCs) for 3D bioprinting. The results showed the SF-dECM constructs had suitable mechanical strength and degradation rate due to the interconnection of SF and dECM through physical crosslinking and entanglement. Moreover, the expression of cartilage chondrogenesis-specific genes was higher than that of SF control construct. This indicated that SF-dECM constructs can promote chondrogenic differentiation of BMSCs and provide a good cartilage repair environment. Wehrle et al. (2019) systematically compared the osteogenic differentiation capacity of four different MSCs isolated from human umbilical cord, adipose tissue, and bone marrow tissue with different hydrogel combinations was also compared. The findings showed that adipose tissue-derived mesenchymal stem cells (AMSCs) displayed the highest osteogenic differentiation potential. A composite hydrogel mixture composed of fibrin, gelatin, hyaluronic acid, and glycerol adjusted with hydroxyapatite showed excellent biocompatibility for AMSC. However, naturally derived biomaterials often result in insufficient mechanical strength, low scaffold fidelity, and loss of osteogenic induction due to the inherent swelling/contraction and biological inertness of most naturally derived biomaterials hydrogels. The graphene oxide-containing bioink was shown to have better bioprintability, scaffold fidelity, cell proliferation, osteogenic differentiation, and ECM mineralization than the pure polymer hydrogel system (Zhang J. et al., 2021).

To scale up 3D bioprinted tissues to clinical size, effective prevascularization strategies are required to provide nutrients required for normal metabolism and remove associated waste by-products. Nulty et al. (2021) developed a bioprinting strategy to engineer prevascular tissues *in vitro* to enhance the ability of blood vessel formation and regeneration in large bone defects *in vivo*. This fibrin-based bioink supported HUVEC sprouting and microvascular network establishment. Three-dimensionally printed PCL scaffolds for prevascularization and implantation into femoral defects in rats showed increased levels of vascularization *in vivo*. This approach could be used to enhance the vascularization of a range of large tissue defects, leading to novel bioprinting therapies.

### Skin

The skin is a complex organ that provides protection, exhibits regulatory functions, and is responsible for communication between the external environment and the organisms within (Perez-Valle et al., 2020). Generally, skin injuries heal spontaneously (Reinke and Sorg, 2012). However, when the skin is severely damaged, it is difficult for it to heal by itself or it cannot heal according to ideal conditions. Artificial bionic skin prepared by 3D bioprinting technology is a better coping strategy (Kim et al., 2020; Jin et al., 2021). Ramakrishnan et al. (2022) optimized the formulation and developed alginate, gelatin, diethylaminoethylcellulose and fibrinogen, a bioink that used the RegenHu 3D Discovery Bioprinter to establish excellent printability, shape fidelity, and cell-laden tissue-equivalent printing. Human primary fibroblasts and keratinocyte-loaded bioprinted constructs exhibited good cell viability. Biomimetic tissue histology was generated after 4 weeks of long-term culture. Specific epidermal-dermal marker expression demonstrating function was evident in immunohistochemical, biochemical and gene expression analyses. Jin et al. (2021) fabricated acellular dermal matrix (ADM) and GelMA bioinks, and proposed a new 3D structure to mimic natural skin, which included 20% GelMA with HaCaTs as an epidermal layer, 1.5% ADM with fibroblasts as the dermis, and 10% GelMA mesh with human umbilical vein endothelial cells as the vascular network and framework. The results showed that the 3D bioprinting skin model could not only promote cell viability and proliferation, but also support epidermis reconstruction *in vitro*. The dECM-based bioinks could be isolated from cell sources associated with skin tissue or stem cells, and they were used to generate 3D skin, through which artificial skin could be efficiently prepared and used to treat wounds (Khoshnood and Zamanian, 2020). Jang et al. (2021) used 3D printing to fabricate structures similar to skin layers using skin-derived dECM, keratinocytes, and fibroblasts. The therapeutic effects of the resulting skin were analyzed using a chimney model that mimics the human wound healing process. The 3D-printed skin substitute was found to exhibit rapid epithelial regeneration and superior tissue regeneration. Promoting vascularization also plays a key role in the treatment of skin wounds, ulcers, and other diseases (Auger et al., 2013). Kim et al. (2018) used skin-derived ECM bioink to print 3D prevascularized skin patches, which could promote wound healing *in vivo*. *In vivo* experimental results showed that endothelial progenitor cells could accelerate wound healing, re-epithelialization, neovascularization, and blood flow when 3D-printed skin patches were combined with adipose-derived stem cells. Sweat glands, as skin appendages, play a crucial role in regulating body temperature in mammals. However, their regenerative potential in response to injury is low. Huang et al. (2016) created a functional *in vitro* cell-borne 3D ECM mimic based on a composite hydrogel of gelatin and sodium alginate. The bioprinted 3D ECM could effectively create a restrictive niche for epidermal progenitor cells, ensure unilateral differentiation into sweat gland cells, and promote the recovery of sweat gland function. Liu et al. (2021) bioprinted MSCs by used bioink alginate-gelatin (Alg-Gel) and investigated the influence of stiffness on MSC differentiation toward sweat glands. Mechanical properties found that higher compressive modulus was associated with the higher Alg-Gel concentrations. MSCs bioprinted by stiffer hydrogels were found to further upregulate the protein and gene expression of sweat gland cell phenotype, function and development of signaling pathways. The results illustrated that the stiffness of Alg-Gel bioink is a potent regulator of MSC differentiation.

### Heart

The heart has a limited ability to regenerate, and the adult heart is the least regenerative organ in the body, which means it is difficult for it to repair itself after damage (Bergmann et al., 2009; Serpooshan and Mahmoudi, 2017). Organ tissue engineering based on 3D bioprinting technology can develop functional tissues and organs, such as *in vivo* grafts to alleviate the shortage of transplanted organs or *in vitro* models for disease mechanism research (Mhashilkar and Atala, 2012). The heart is a vital organ and comprises multiple cells, including fibroblasts, endothelial cells, cardiomyocytes, smooth muscle cells, and pacemaker cells, all of which are structurally organized in a mixture of ECM materials. Therefore, 3D bioprinting of cardiac tissue often uses multicomponent bioinks. Roche et al. (2021) 3D bioprinted cardiac patches using alginate/gelatin (Alg/Gel) hydrogels and cardiac endothelial cells. The cardiac patchs presented endothelial cell networks, durable structure, and contractile function between 14 and 28 days in culture. The findings have the potential to directly translate *in vitro* testing of bioprinted cardiac patches into *in vivo* applications for cardiac regeneration. Gaetani et al. (2015) used hyaluronic acid and gelatin 3D-printed patches containing human cardiac-derived progenitor cells and transplanted these patches into a mouse model of myocardial infarction, showing good cell survival/transplantation and increased markers of cardiac and vascular differentiation. The cardiac function improved after cardiac patch. The cardiac-derived dECM was functionally and structurally similar to native tissue, and this bioink exhibited a high degree of differentiation and maturation of cardiac tissue and a suitable tissue-mimicking microenvironment. Therefore, Noor et al. (2019) used bioinks containing dECM hydrogels in combination with cardiomyocytes to print whole hearts with major blood vessels, demonstrating the potential of the approach for organ replacement after failure or for drug screening in an appropriate anatomical structure. The low mechanical stability of dECM prevents its use in bioprinting applications by itself. Basara et al. (2021) mixed GelMA and GelMA-MeHA hydrogels with decellularized human cardiac ECM (dhECM) to create cardiac tissue-like structures. Compared with GelMA-dhECM hydrogel, the mechanical properties of composite GelMA-MeHA-dhECM hydrogel were improved by an order of magnitude. These hydrogels were compatible with human-induced pluripotent stem cell-derived cardiomyocytes and human cardiac fibroblasts, with the observation of printed structures with striated sarcomere α-actin and connexin 43 expression and tissue-like beating. The order-of-magnitude difference between the elastic moduli of these hydrogel composites provides applications for *in vitro* modeling of myocardial infarction boundaries.

### Liver

The liver is the key metabolic organ of the body, and it has good self-regeneration ability. However, severe and chronic damage will affect its ability to regenerate properly (Ashammakhi et al., 2019). Zhong et al. (2016) used a bioink containing type I collagen and chitosan to fabricate a 3D hydrogel scaffold, which was loaded with L02 (cell line HL-7702) cells and then transplanted into the liver of mice with liver damage. The 3D hydrogel scaffolds did not affect cell viability after weeks of inspection. HE staining showed clear liver tissue, and immunohistochemistry for cKit and CK18 in the transplanted tissue was positive. The scaffold could be used for reconstruction of liver tissue. In another study, primary human hepatocytes and hepatic stellate cells were 3D-printed with bioinks containing methacrylated type I collagen and thiolated hyaluronic acid to 3D-print liver tissue structures. The bioink sufficiently allowed implementation as a supporting hydrogel for hepatocytes, which were able to remain viable and respond appropriately to drug treatment for 2 weeks. Liver dECM can enhance cell viability (Mazzocchi et al., 2018). Mao Q. et al. (2020) developed a liver-specific bioink by combining liver decellularized extracellular matrix with GelMA to print liver microtissues after encapsulation of human-induced hepatocytes (hiHep cells). The liver dECM was found to improve not only the printability but also the hiHep cell viability of bioinks. This would be a potential liver tissue engineering product that could help restore liver function.

### Blood Vessel

Traditional 3D printing methods have limited ability to construct vascular features (Dolati et al., 2014). New bioprinting techniques show great potential in printing blood vessels. For example, Li et al. (2022) connected a coaxial microfluidic system to a 3D printer and used MeHA/alginate bioink to customize microvessels with personalized shapes. There has also been considerable success using sacrificial materials to reduce the diameter of vascular channels. Kolesky et al. (2014) used Pluronic F127 to print small vascular channels with a diameter of 45 μm encapsulated with GelMA ink and irradiated with UV light to crosslink the GelMA matrix. The entire structure was finally cooled to 4°C to liquefy the Pluronic F127 for removal, leaving open, interconnected microchannels to provide the desired embedded vascular network. In addition, gelatin (Lee et al., 2014), carbohydrate glass (Miller et al., 2012), alginate (Contessi Negrini et al., 2019), and others are also used as sacrificial materials to prepare blood vessels. These various studies demonstrate that printing blood vessels using sacrificial techniques not only modulates the pre-patterning of vascular features but also provides a basis for the fabrication of large tissue structures (Aljohani et al., 2018).

### Neuronal Tissues

The natural regeneration potential of neurons is limited, and with the rapid development of 3D bioprinting technology, bioprinting neuronal tissue is a very important breakthrough. Bioprinting of neuronal tissue works in two ways: one is to print new neuronal tissue and the other is to enhance the innervation of an already engineered tissue structure, which, after implantation at the target site, integrates with the host nervous system to reveal its effect (Mandrycky et al., 2016). Hirano et al. (2021) developed an induced pluripotent stem cell (iPSC)/sensory neuron (SN)/loaded gelatin bioink. After printing on a laminin-coated substrate using extrusion-based bioprinting, the iPSC-SNs were seeded into the hollow microchannels created by sacrificial gelatin ink printed in the gelatin methacryloyl supporting bath, thereby demonstrating controllability over axon guidance in curved lines up to several tens of centimeters in length on 2D substrates and in straight microchannels in 3D matrices. This approach integrated sensitive SN networks into engineered skin equivalents, regenerative skin implants, and enhanced somatosensory prostheses to regenerate sensitive functions by connecting the host neuronal system in the injured area. In the bioink containing HA/gelatin/heparan sulfate/novel chitosan developed by Guan et al. (2013), the printed scaffold supported the adhesion and long-term growth of naive neural stem and progenitor cells, providing a new option for neural tissue engineering applications. In addition, gelatin can also create a favorable microenvironment for neuronal axon regeneration and synaptogenesis for spinal cord injury repair (Liu et al., 2019).

### Disease Models

Cell culture models have played an important role in enhancing our understanding of disease development and progression. However, it is well-recognized that those systems fail to accurately represent the disease ecosystems or mimic in a precise manner the cellular interactions that take place in the disease tissues (Sitarski et al., 2018; Bae et al., 2020; Gao et al., 2021). The use of 3D-bioprinted tumors is increasing in areas such as tumor biology, migration, invasion, and metastasis and high-throughput drug screening and validation (Sánchez-Salazar et al., 2021). For example, Herrada-Manchón et al. (2021) used an optimized collagen/alginate/gelatin hydrogel and optimized printing parameters to bioprint renal cancer cells. In this context, cells were viable, proliferated for long time periods of time and form long and thin TNT-like structures that are used as channels for the long-distance cell-to-cell transfer of mitochondria. This provides a novel alternative tool for studying the functional relevance of TNT-like structures in tumorigenesis and anticancer drug susceptibility in a highly controlled and reproducible tumor microenvironment. In the research of liver cancer, Ma et al. (2018) developed a photo-crosslinkable liver dECM and a light-based rapid 3D bioprinting process. The hepatic dECM scaffolds printed in this way stably mimicked the clinically relevant mechanical properties of cirrhotic liver tissue. This *in vitro* dECM-based 3D biomimetic liver platform could be used to study the behavior of various liver cancer cells in specific fibrotic settings to help elucidate disease mechanisms in biological research and applications in preclinical drug screening. In addition, new printing methods are constantly emerging. Maloney et al. (2020) developed an immersion bioprinting method based on a hydrogel bioink composed of hyaluronic acid and collagen. This method could be used for tumor model establishment, drug development, and other applications.

## Conclusion

Using 3D bioprinting and ECM-based bioinks to mimic the structure of native tissues provides a new direction for tissue regeneration and organ construction. Although much work has been done, many obstacles preventing the development of this technology still remain. There is no standard method to extract native ECM, with poor batch-to-batch reproducibility. Moreover, improving the resolution of 3D bioprinting to ensure cell viability and function *in vitro* and *in vivo* is still a challenge. Hence, extensive research is still needed in both bioink and printing technology, and especially in the combination of interdisciplinary methods, to make 3D bioprinting a powerful tool in the biomedical system and to transform the technology into clinical practice for fostering a revolution in people’s health and lives.

## References

[B1] Abaci A. Guvendiren M. (2020). Designing Decellularized Extracellular Matrix‐Based Bioinks for 3D Bioprinting. Adv. Healthc. Mat. 9, 2000734. 10.1002/adhm.202000734 32691980

[B2] Additive Manufacturing Research Group (2019). The 7 Categories of Additive Manufacturing. Loughborough University. [Online] Available: https://www.lboro.ac.uk/research/amrg/about/the7categoriesofadditivemanufacturing/ (Accessed Feb 28, 2022).

[B3] Aghmiuni A. I. Khiavi A. A. (2017). Medicinal Plants to Calm and Treat Psoriasis Disease. London: IntechOpen.

[B4] Aljohani W. Ullah M. W. Zhang X. Yang G. (2018). Bioprinting and its Applications in Tissue Engineering and Regenerative Medicine. Int. J. Biol. Macromol. 107, 261–275. 10.1016/j.ijbiomac.2017.08.171 28870749

[B5] Antich C. De Vicente J. Jiménez G. Chocarro C. Carrillo E. Montañez E. (2020). Bio-inspired Hydrogel Composed of Hyaluronic Acid and Alginate as a Potential Bioink for 3D Bioprinting of Articular Cartilage Engineering Constructs. Acta Biomater. 106, 114–123. 10.1016/j.actbio.2020.01.046 32027992

[B6] Arab W. Rauf S. Al-Harbi O. Hauser C. (2018). Novel Ultrashort Self-Assembling Peptide Bioinks for 3D Culture of Muscle Myoblast Cells. Int. J. Bioprint 4, 129. 10.18063/ijb.v4i1.129 33102913 PMC7582005

[B7] Ashammakhi N. Ahadian S. Xu C. Montazerian H. Ko H. Nasiri R. (2019). Bioinks and Bioprinting Technologies to Make Heterogeneous and Biomimetic Tissue Constructs. Mater. Today Bio 1, 100008. 10.1016/j.mtbio.2019.100008 PMC7061634 32159140

[B8] Attalla R. Ling C. S. N. Selvaganapathy P. R. (2018). Silicon Carbide Nanoparticles as an Effective Bioadhesive to Bond Collagen Containing Composite Gel Layers for Tissue Engineering Applications. Adv. Healthc. Mat. 7, 1701385. 10.1002/adhm.201701385 29360239

[B9] Auger F. A. Gibot L. Lacroix D. (2013). The Pivotal Role of Vascularization in Tissue Engineering. Annu. Rev. Biomed. Eng. 15, 177–200. 10.1146/annurev-bioeng-071812-152428 23642245

[B10] Bae M. Yi H.-G. Jang J. Cho D.-W. (2020). Microphysiological Systems for Neurodegenerative Diseases in Central Nervous System. Micromachines 11, 855. 10.3390/mi11090855 PMC7570039 32947879

[B11] Banche-Niclot F. Montalbano G. Fiorilli S. Vitale-Brovarone C. (2021). PEG-coated Large Mesoporous Silicas as Smart Platform for Protein Delivery and Their Use in a Collagen-Based Formulation for 3D Printing. Ijms 22, 1718. 10.3390/ijms22041718 33572076 PMC7914545

[B12] Basara G. Ozcebe S. G. Ellis B. W. Zorlutuna P. (2021). Tunable Human Myocardium Derived Decellularized Extracellular Matrix for 3D Bioprinting and Cardiac Tissue Engineering. Gels 7, 70. 10.3390/gels7020070 34208210 PMC8293197

[B13] Bendtsen S. T. Quinnell S. P. Wei M. (2017). Development of a Novel Alginate‐polyvinyl Alcohol‐hydroxyapatite Hydrogel for 3D Bioprinting Bone Tissue Engineered Scaffolds. J. Biomed. Mat. Res. 105, 1457–1468. 10.1002/jbm.a.36036 28187519

[B14] Bergmann O. Bhardwaj R. D. Bernard S. Zdunek S. Barnabé-Heider F. Walsh S. (2009). Evidence for Cardiomyocyte Renewal in Humans. Science 324, 98–102. 10.1126/science.1164680 19342590 PMC2991140

[B15] Bertuola M. Aráoz B. Gilabert U. Gonzalez-Wusener A. Pérez-Recalde M. Arregui C. O. (2021). Gelatin-alginate-hyaluronic Acid Inks for 3D Printing: Effects of Bioglass Addition on Printability, Rheology and Scaffold Tensile Modulus. J. Mat. Sci. 56, 15327–15343. 10.1007/s10853-021-06250-0

[B16] Billiet T. Gevaert E. De Schryver T. Cornelissen M. Dubruel P. (2014). The 3D Printing of Gelatin Methacrylamide Cell-Laden Tissue-Engineered Constructs with High Cell Viability. Biomaterials 35, 49–62. 10.1016/j.biomaterials.2013.09.078 24112804

[B17] Blyweert P. Nicolas V. Fierro V. Celzard A. (2021). 3D Printing of Carbon-Based Materials: a Review. Carbon 183, 449–485. 10.1016/j.carbon.2021.07.036

[B18] Cakmak A. M. Unal S. Sahin A. Oktar F. N. Sengor M. Ekren N. (2020). 3D Printed Polycaprolactone/Gelatin/Bacterial Cellulose/Hydroxyapatite Composite Scaffold for Bone Tissue Engineering. Polymers 12, 1962. 10.3390/polym12091962 PMC7570222 32872547

[B19] Cao Y. Cheng P. Sang S. Xiang C. An Y. Wei X. (2021). 3D Printed PCL/GelMA Biphasic Scaffold Boosts Cartilage Regeneration Using Co-culture of Mesenchymal Stem Cells and Chondrocytes: *In Vivo* Study. Mater. Des. 210, 110065. 10.1016/j.matdes.2021.110065

[B20] Carew R. M. Errickson D. (2020). An Overview of 3D Printing in Forensic Science: The Tangible Third‐Dimension. J. Forensic. Sci. 65, 1752–1760. 10.1111/1556-4029.14442 32401341

[B21] Chatterjee K. Ghosh T. K. (2020). 3D Printing of Textiles: Potential Roadmap to Printing with Fibers. Adv. Mat. 32, 1902086. 10.1002/adma.201902086 31788860

[B22] Chen M. Li Y. Liu S. Feng Z. Wang H. Yang D. (2021). Hierarchical Macro-Microporous WPU-ECM Scaffolds Combined with Microfracture Promote *In Situ* Articular Cartilage Regeneration in Rabbits. Bioact. Mater. 6, 1932–1944. 10.1016/j.bioactmat.2020.12.009 33426368 PMC7772526

[B23] Chivers P. R. A. Smith D. K. (2019). Shaping and Structuring Supramolecular Gels. Nat. Rev. Mat. 4, 463–478. 10.1038/s41578-019-0111-6

[B24] Choudhury D. Tun H. W. Wang T. Naing M. W. (2018). Organ-Derived Decellularized Extracellular Matrix: A Game Changer for Bioink Manufacturing? Trends Biotechnol. 36, 787–805. 10.1016/j.tibtech.2018.03.003 29678431

[B25] Contessi Negrini N. Bonnetier M. Giatsidis G. Orgill D. P. Farè S. Marelli B. (2019). Tissue-mimicking Gelatin Scaffolds by Alginate Sacrificial Templates for Adipose Tissue Engineering. Acta Biomater. 87, 61–75. 10.1016/j.actbio.2019.01.018 30654214

[B26] Cunniffe G. M. Gonzalez-Fernandez T. Daly A. Sathy B. N. Jeon O. Alsberg E. (2017). Three-Dimensional Bioprinting of Polycaprolactone Reinforced Gene Activated Bioinks for Bone Tissue Engineering. Tissue Eng. Part A 23, 891–900. 10.1089/ten.tea.2016.0498 28806146

[B27] Das A. K. Gavel P. K. (2020). Low Molecular Weight Self-Assembling Peptide-Based Materials for Cell Culture, Antimicrobial, Anti-inflammatory, Wound Healing, Anticancer, Drug Delivery, Bioimaging and 3D Bioprinting Applications. Soft Matter 16, 10065–10095. 10.1039/D0SM01136C 33073836

[B28] Das S. Basu B. (2022). Extrusion‐based 3D Printing of Gelatin Methacryloyl with Nanocrystalline Hydroxyapatite. Int. J. Appl. Ceram. Tech. 19, 924–938. 10.1111/ijac.13885

[B29] Demirtaş T. T. Irmak G. Gümüşderelioğlu M. (2017). A Bioprintable Form of Chitosan Hydrogel for Bone Tissue Engineering. Biofabrication 9, 035003. 10.1088/1758-5090/aa7b1d 28639943

[B30] Diamantides N. Wang L. Pruiksma T. Siemiatkoski J. Dugopolski C. Shortkroff S. (2017). Correlating Rheological Properties and Printability of Collagen Bioinks: the Effects of Riboflavin Photocrosslinking and pH. Biofabrication 9, 034102. 10.1088/1758-5090/aa780f 28677597

[B31] Diloksumpan P. De Ruijter M. Castilho M. Gbureck U. Vermonden T. Van Weeren P. R. (2020). Combining Multi-Scale 3D Printing Technologies to Engineer Reinforced Hydrogel-Ceramic Interfaces. Biofabrication 12, 025014. 10.1088/1758-5090/ab69d9 31918421 PMC7116207

[B32] Dolati F. Yu Y. Zhang Y. Jesus A. M. D. Sander E. A. Ozbolat I. T. (2014). In Vitroevaluation of Carbon-Nanotube-Reinforced Bioprintable Vascular Conduits. Nanotechnology 25, 145101. 10.1088/0957-4484/25/14/145101 24632802 PMC4281171

[B33] Drzewiecki K. E. Malavade J. N. Ahmed I. Lowe C. J. Shreiber D. I. (2017). A Thermoreversible, Photocrosslinkable Collagen Bio-Ink for Free-form Fabrication of Scaffolds for Regenerative Medicine. Technology 05, 185–195. 10.1142/s2339547817500091 PMC5845803 29541655

[B34] Duan B. Hockaday L. A. Kang K. H. Butcher J. T. (2013). 3D Bioprinting of Heterogeneous Aortic Valve Conduits with Alginate/gelatin Hydrogels. J. Biomed. Mat. Res. 101A, 1255–1264. 10.1002/jbm.a.34420 PMC3694360 23015540

[B35] Dzobo K. Motaung K. S. C. M. Adesida A. (2019). Recent Trends in Decellularized Extracellular Matrix Bioinks for 3D Printing: An Updated Review. Ijms 20, 4628. 10.3390/ijms20184628 PMC6788195 31540457

[B36] Elomaa L. Keshi E. Sauer I. M. Weinhart M. (2020). Development of GelMA/PCL and dECM/PCL Resins for 3D Printing of Acellular *In Vitro* Tissue Scaffolds by Stereolithography. Mater. Sci. Eng. C 112, 110958. 10.1016/j.msec.2020.110958 32409091

[B37] Fermani M. Platania V. Kavasi R.-M. Karavasili C. Zgouro P. Fatouros D. (2021). 3D-Printed Scaffolds from Alginate/Methyl Cellulose/Trimethyl Chitosan/Silicate Glasses for Bone Tissue Engineering. Appl. Sci. 11, 8677. 10.3390/app11188677

[B38] Gaetani R. Feyen D. A. M. Verhage V. Slaats R. Messina E. Christman K. L. (2015). Epicardial Application of Cardiac Progenitor Cells in a 3D-Printed Gelatin/hyaluronic Acid Patch Preserves Cardiac Function after Myocardial Infarction. Biomaterials 61, 339–348. 10.1016/j.biomaterials.2015.05.005 26043062

[B39] Gao G. Park W. Kim B. S. Ahn M. Chae S. Cho W. W. (2021). Construction of a Novel *In Vitro* Atherosclerotic Model from Geometry‐Tunable Artery Equivalents Engineered via in‐Bath Coaxial Cell Printing. Adv. Funct. Mat. 31, 2008878. 10.1002/adfm.202008878

[B40] Gattazzo F. Urciuolo A. Bonaldo P. (2014). Extracellular Matrix: a Dynamic Microenvironment for Stem Cell Niche. Biochimica Biophysica Acta (BBA) - General Subj. 1840, 2506–2519. 10.1016/j.bbagen.2014.01.010 PMC4081568 24418517

[B41] Gentile C. G. Sharma P. Ashton A. W. Jackson C. Xue M. Gentile C. (2021). Printability, Durability, Contractility and Vascular Network Formation in 3D Bioprinted Cardiac Endothelial Cells Using Alginate-Gelatin Hydrogels. fbioe 9, 636257. 10.3389/fbioe.2021.636257 PMC7968457 33748085

[B42] Ghalayini S. Susapto H. H. Hall S. Kahin K. Hauser C. (2019). Preparation and Printability of Ultrashort Self-Assembling Peptide Nanoparticles. Int. J. Bioprint 5, 239. 10.18063/ijb.v5i2.239 32596541 PMC7294693

[B43] Ghorbani F. Li D. Zhong Z. Sahranavard M. Qian Z. Ni S. (2021). Bioprinting a Cell-Laden Matrix for Bone Regeneration: A Focused Review. J. Appl. Polym. Sci. 138, 49888. 10.1002/app.49888

[B44] Gilpin A. Yang Y. (2017). Decellularization Strategies for Regenerative Medicine: From Processing Techniques to Applications. BioMed Res. Int. 2017, 1–13. 10.1155/2017/9831534 PMC5429943 28540307

[B45] Guan S. Zhang X.-L. Lin X.-M. Liu T.-Q. Ma X.-H. Cui Z.-F. (2013). Chitosan/gelatin Porous Scaffolds Containing Hyaluronic Acid and Heparan Sulfate for Neural Tissue Engineering. J. Biomaterials Sci. Polym. Ed. 24, 999–1014. 10.1080/09205063.2012.731374 23647254

[B46] Guvendiren M. Molde J. Soares R. M. D. Kohn J. (2016). Designing Biomaterials for 3D Printing. ACS Biomater. Sci. Eng. 2, 1679–1693. 10.1021/acsbiomaterials.6b00121 28025653 PMC5181796

[B47] Han W. Singh N. K. Kim J. J. Kim H. Kim B. S. Park J. Y. (2019). Directed Differential Behaviors of Multipotent Adult Stem Cells from Decellularized Tissue/organ Extracellular Matrix Bioinks. Biomaterials 224, 119496. 10.1016/j.biomaterials.2019.119496 31557592

[B48] Herrada-Manchón H. Celada L. Rodríguez-González D. Alejandro Fernández M. Aguilar E. Chiara M.-D. (2021). Three-dimensional Bioprinted Cancer Models: A Powerful Platform for Investigating Tunneling Nanotube-like Cell Structures in Complex Microenvironments. Mater. Sci. Eng. C 128, 112357. 10.1016/j.msec.2021.112357 34474904

[B49] Hirano M. Huang Y. Vela Jarquin D. De la Garza Hernández R. L. Jodat Y. A. Luna Cerón E. (2021). 3D Bioprinted Human iPSC-Derived Somatosensory Constructs with Functional and Highly Purified Sensory Neuron Networks. Biofabrication 13, 035046. 10.1088/1758-5090/abff11 33962404

[B50] Hospodiuk M. Dey M. Sosnoski D. Ozbolat I. T. (2017). The Bioink: A Comprehensive Review on Bioprintable Materials. Biotechnol. Adv. 35, 217–239. 10.1016/j.biotechadv.2016.12.006 28057483

[B51] Hsieh C.-T. Hsu S.-h. (2019). Double-Network Polyurethane-Gelatin Hydrogel with Tunable Modulus for High-Resolution 3D Bioprinting. ACS Appl. Mat. Interfaces 11, 32746–32757. 10.1021/acsami.9b10784 31407899

[B52] Huang J. Huang Z. Liang Y. Yuan W. Bian L. Duan L. (2021). 3D Printed Gelatin/hydroxyapatite Scaffolds for Stem Cell Chondrogenic Differentiation and Articular Cartilage Repair. Biomater. Sci. 9, 2620–2630. 10.1039/d0bm02103b 33595025

[B53] Huang S. Yao B. Xie J. Fu X. (2016). 3D Bioprinted Extracellular Matrix Mimics Facilitate Directed Differentiation of Epithelial Progenitors for Sweat Gland Regeneration. Acta Biomater. 32, 170–177. 10.1016/j.actbio.2015.12.039 26747979

[B54] Hubs D. (2018). What Is 3D Printing? the Definitive Guide. [Online]. Available: https://www.3dhubs.com/guides/3d-printing/ (Accessed Jan15, 2022).

[B55] Isaacson A. Swioklo S. Connon C. J. (2018). 3D Bioprinting of a Corneal Stroma Equivalent. Exp. Eye Res. 173, 188–193. 10.1016/j.exer.2018.05.010 29772228 PMC6083436

[B56] Jang K.-S. Park S.-J. Choi J.-J. Kim H.-N. Shim K.-M. Kim M.-J. (2021). Therapeutic Efficacy of Artificial Skin Produced by 3D Bioprinting. Materials 14, 5177. 10.3390/ma14185177 34576409 PMC8467964

[B57] Jeon O. Song S. J. Lee K.-J. Park M. H. Lee S.-H. Hahn S. K. (2007). Mechanical Properties and Degradation Behaviors of Hyaluronic Acid Hydrogels Cross-Linked at Various Cross-Linking Densities. Carbohydr. Polym. 70, 251–257. 10.1016/j.carbpol.2007.04.002

[B58] Jian H. Wang M. Dong Q. Li J. Wang A. Li X. (2019). Dipeptide Self-Assembled Hydrogels with Tunable Mechanical Properties and Degradability for 3D Bioprinting. ACS Appl. Mat. Interfaces 11, 46419–46426. 10.1021/acsami.9b13905 31769283

[B59] Jiang J. Liu X. Chen H. Dai C. Niu X. Dai L. (2020). 3D Printing Collagen/heparin Sulfate Scaffolds Boost Neural Network Reconstruction and Motor Function Recovery after Traumatic Brain Injury in Canine. Biomater. Sci. 8, 6362–6374. 10.1039/D0BM01116A 33026366

[B60] Jin R. Cui Y. Chen H. Zhang Z. Weng T. Xia S. (2021). Three-dimensional Bioprinting of a Full-Thickness Functional Skin Model Using Acellular Dermal Matrix and Gelatin Methacrylamide Bioink. Acta Biomater. 131, 248–261. 10.1016/j.actbio.2021.07.012 34265473

[B61] Kalkandelen C. Ulag S. Ozbek B. Eroglu G. O. Ozerkan D. Kuruca S. E. (2019). 3D Printing of Gelatine/Alginate/β‐Tricalcium Phosphate Composite Constructs for Bone Tissue Engineering. ChemistrySelect 4, 12032–12036. 10.1002/slct.201902878

[B62] Kesti M. Müller M. Becher J. Schnabelrauch M. D’Este M. Eglin D. (2015). A Versatile Bioink for Three-Dimensional Printing of Cellular Scaffolds Based on Thermally and Photo-Triggered Tandem Gelation. Acta Biomater. 11, 162–172. 10.1016/j.actbio.2014.09.033 25260606

[B63] Khoshnood N. Zamanian A. (2020). Decellularized Extracellular Matrix Bioinks and Their Application in Skin Tissue Engineering. Bioprinting 20, e00095. 10.1016/j.bprint.2020.e00095

[B64] Kim B. S. Das S. Jang J. Cho D.-W. (2020). Decellularized Extracellular Matrix-Based Bioinks for Engineering Tissue- and Organ-specific Microenvironments. Chem. Rev. 120, 10608–10661. 10.1021/acs.chemrev.9b00808 32786425

[B65] Kim B. S. Kwon Y. W. Kong J.-S. Park G. T. Gao G. Han W. (2018). 3D Cell Printing of *In Vitro* Stabilized Skin Model and *In Vivo* Pre-vascularized Skin Patch Using Tissue-specific Extracellular Matrix Bioink: A Step towards Advanced Skin Tissue Engineering. Biomaterials 168, 38–53. 10.1016/j.biomaterials.2018.03.040 29614431

[B66] Kim H. S. Kim C. Lee K. Y. (2022). Three‐dimensional Bioprinting of Polysaccharide‐based Self‐healing Hydrogels with Dual Cross‐linking. J. Biomed. Mater. Res. 110, 761–772. 10.1002/jbm.a.37325 34708518

[B67] Kim J.-W. Han Y.-S. Lee H.-M. Kim J.-K. Kim Y.-J. (2021). Effect of Morphological Characteristics and Biomineralization of 3D-Printed Gelatin/Hyaluronic Acid/Hydroxyapatite Composite Scaffolds on Bone Tissue Regeneration. Ijms 22, 6794. 10.3390/ijms22136794 34202759 PMC8267715

[B68] Kim H. Kang B. Cui X. Lee S. H. Lee K. Cho D. W. (2021). Light‐Activated Decellularized Extracellular Matrix‐Based Bioinks for Volumetric Tissue Analogs at the Centimeter Scale. Adv. Funct. Mater. 31, 2011252. 10.1002/adfm.202011252

[B69] Kim Y. B. Lee H. Kim G. H. (2016). Strategy to Achieve Highly Porous/Biocompatible Macroscale Cell Blocks, Using a Collagen/Genipin-Bioink and an Optimal 3D Printing Process. ACS Appl. Mat. Interfaces 8, 32230–32240. 10.1021/acsami.6b11669 27933843

[B70] Kim Y. S. Majid M. Melchiorri A. J. Mikos A. G. (2019). Applications of Decellularized Extracellular Matrix in Bone and Cartilage Tissue Engineering. Bioeng. Transl. Med. 4, 83–95. 10.1002/btm2.10110 30680321 PMC6336671

[B71] Knudson C. B. (2003). Hyaluronan and CD44: Strategic Players for Cell-Matrix Interactions during Chondrogenesis and Matrix Assembly. Birth Defect Res. C 69, 174–196. 10.1002/bdrc.10013 12955860

[B72] Kolesky D. B. Truby R. L. Gladman A. S. Busbee T. A. Homan K. A. Lewis J. A. (2014). 3D Bioprinting of Vascularized, Heterogeneous Cell-Laden Tissue Constructs. Adv. Mat. 26, 3124–3130. 10.1002/adma.201305506 24550124

[B73] Köpf M. Campos D. F. D. Blaeser A. Sen K. S. Fischer H. (2016). A Tailored Three-Dimensionally Printable Agarose-Collagen Blend Allows Encapsulation, Spreading, and Attachment of Human Umbilical Artery Smooth Muscle Cells. Biofabrication 8, 025011. 10.1088/1758-5090/8/2/025011 27205890

[B74] Kreimendahl F. Köpf M. Thiebes A. L. Duarte Campos D. F. Blaeser A. Schmitz-Rode T. (2017). Three-Dimensional Printing and Angiogenesis: Tailored Agarose-type I Collagen Blends Comprise Three-Dimensional Printability and Angiogenesis Potential for Tissue-Engineered Substitutes. Tissue Eng. Part C. Methods 23, 604–615. 10.1089/ten.TEC.2017.0234 28826357

[B75] Kulkarni P. Marsan A. Dutta D. (2000). A Review of Process Planning Techniques in Layered Manufacturing. Rapid Prototyp. J. 6, 18–35. 10.1108/13552540010309859

[B76] Kupfer M. E. Lin W.-H. Ravikumar V. Qiu K. Wang L. Gao L. (2020). *In Situ* Expansion, Differentiation, and Electromechanical Coupling of Human Cardiac Muscle in a 3D Bioprinted, Chambered Organoid. Circ. Res. 127, 207–224. 10.1161/circresaha.119.316155 32228120 PMC8210857

[B77] Lee A. Hudson A. R. Shiwarski D. J. Tashman J. W. Hinton T. J. Yerneni S. (2019). 3D Bioprinting of Collagen to Rebuild Components of the Human Heart. Science 365, 482–487. 10.1126/science.aav9051 31371612

[B78] Lee V. K. Kim D. Y. Ngo H. Lee Y. Seo L. Yoo S.-S. (2014). Creating Perfused Functional Vascular Channels Using 3D Bio-Printing Technology. Biomaterials 35, 8092–8102. 10.1016/j.biomaterials.2014.05.083 24965886 PMC4112057

[B79] Li C. Han X. Ma Z. Jie T. Wang J. Deng L. (2022). Engineered Customizable Microvessels for Progressive Vascularization in Large Regenerative Implants. Adv. Healthc. Mater. 11, 2101836. 10.1002/adhm.202101836 34797037

[B80] Li L. Qin S. Peng J. Chen A. Nie Y. Liu T. (2020). Engineering Gelatin-Based Alginate/carbon Nanotubes Blend Bioink for Direct 3D Printing of Vessel Constructs. Int. J. Biol. Macromol. 145, 262–271. 10.1016/j.ijbiomac.2019.12.174 31870866

[B81] Li X. Wang Y. Wang Z. Qi Y. Li L. Zhang P. (2018). Composite PLA/PEG/nHA/Dexamethasone Scaffold Prepared by 3D Printing for Bone Regeneration. Macromol. Biosci. 18, 1800068. 10.1002/mabi.201800068 29687630

[B82] Liu W. Heinrich M. A. Zhou Y. Akpek A. Hu N. Liu X. (2017). Extrusion Bioprinting of Shear‐Thinning Gelatin Methacryloyl Bioinks. Adv. Healthc. Mat. 6, 1601451. 10.1002/adhm.201601451 PMC5545786 28464555

[B83] Liu Y. Li J. Yao B. Wang Y. Wang R. Yang S. (2021). The Stiffness of Hydrogel-Based Bioink Impacts Mesenchymal Stem Cells Differentiation toward Sweat Glands in 3D-Bioprinted Matrix. Mater. Sci. Eng. C 118, 111387. 10.1016/j.msec.2020.111387 33254993

[B84] Loo Y. Lakshmanan A. Ni M. Toh L. L. Wang S. Hauser C. A. E. (2015). Peptide Bioink: Self-Assembling Nanofibrous Scaffolds for Three-Dimensional Organotypic Cultures. Nano Lett. 15, 6919–6925. 10.1021/acs.nanolett.5b02859 26214046

[B85] Lu H. Pan X. Hu M. Zhang J. Yu Y. Hu X. (2021). Fabrication of Graphene/gelatin/chitosan/tricalcium Phosphate 3D Printed Scaffolds for Bone Tissue Regeneration Applications. Appl. Nanosci. 11, 335–346. 10.1007/s13204-020-01615-4

[B86] Lu P. Takai K. Weaver V. M. Werb Z. (2011). Extracellular Matrix Degradation and Remodeling in Development and Disease. Cold Spring Harb. Perspect. Biol. 3, a005058. 10.1101/cshperspect.a005058 21917992 PMC3225943

[B87] Ma D. Gao R. Li M. Qiu J. (2022). Mechanical and Medical Imaging Properties of 3D‐printed Materials as Tissue Equivalent Materials. J. Appl. Clin. Med. Phys. 23, e13495. 10.1002/acm2.13495 34878729 PMC8833282

[B88] Ma X. Yu C. Wang P. Xu W. Wan X. Lai C. S. E. (2018). Rapid 3D Bioprinting of Decellularized Extracellular Matrix with Regionally Varied Mechanical Properties and Biomimetic Microarchitecture. Biomaterials 185, 310–321. 10.1016/j.biomaterials.2018.09.026 30265900 PMC6186504

[B89] Maloney E. Clark C. Sivakumar H. Yoo K. Aleman J. Rajan S. A. P. (2020). Immersion Bioprinting of Tumor Organoids in Multi-Well Plates for Increasing Chemotherapy Screening Throughput. Micromachines 11, 208. 10.3390/mi11020208 PMC7074680 32085455

[B90] Mandrycky C. Wang Z. Kim K. Kim D.-H. (2016). 3D Bioprinting for Engineering Complex Tissues. Biotechnol. Adv. 34, 422–434. 10.1016/j.biotechadv.2015.12.011 26724184 PMC4879088

[B91] Mao H. Yang L. Zhu H. Wu L. Ji P. Yang J. (2020). Recent Advances and Challenges in Materials for 3D Bioprinting. Prog. Nat. Sci. Mater. Int. 30, 618–634. 10.1016/j.pnsc.2020.09.015

[B92] Mao Q. Wang Y. Li Y. Juengpanich S. Li W. Chen M. (2020). Fabrication of Liver Microtissue with Liver Decellularized Extracellular Matrix (dECM) Bioink by Digital Light Processing (DLP) Bioprinting. Mater. Sci. Eng. C 109, 110625. 10.1016/j.msec.2020.110625 32228893

[B93] Matai I. Kaur G. Seyedsalehi A. Mcclinton A. Laurencin C. T. (2020). Progress in 3D Bioprinting Technology for Tissue/organ Regenerative Engineering. Biomaterials 226, 119536. 10.1016/j.biomaterials.2019.119536 31648135

[B94] Mazzocchi A. Devarasetty M. Huntwork R. Soker S. Skardal A. (2018). Optimization of Collagen Type I-Hyaluronan Hybrid Bioink for 3D Bioprinted Liver Microenvironments. Biofabrication 11, 015003. 10.1088/1758-5090/aae543 30270846 PMC8008502

[B95] Meyer M. (2019). Processing of Collagen Based Biomaterials and the Resulting Materials Properties. Biomed. Eng. OnLine 18, 24. 10.1186/s12938-019-0647-0 30885217 PMC6423854

[B96] Miller J. S. Stevens K. R. Yang M. T. Baker B. M. Nguyen D. H. Cohen D. M. (2012). Rapid Casting of Patterned Vascular Networks for Perfusable Engineered Three-Dimensional Tissues. Nat. Mat. 11, 768–774. 10.1038/nmat3357 PMC3586565 22751181

[B97] M. Mhashilkar A. Atala A. (2012). Advent and Maturation of Regenerative Medicine. Cscr 7, 430–445. 10.2174/157488812804484657 23176292

[B98] Munaz A. Vadivelu R. K. St. John J. Barton M. Kamble H. Nguyen N.-T. (2016). Three-dimensional Printing of Biological Matters. J. Sci. Adv. Mater. Devices 1, 1–17. 10.1016/j.jsamd.2016.04.001

[B99] Nedunchezian S. Banerjee P. Lee C.-Y. Lee S.-S. Lin C.-W. Wu C.-W. (2021). Generating Adipose Stem Cell-Laden Hyaluronic Acid-Based Scaffolds Using 3D Bioprinting via the Double Crosslinked Strategy for Chondrogenesis. Mater. Sci. Eng. C 124, 112072. 10.1016/j.msec.2021.112072 33947564

[B100] Nelson M. Li S. Page S. J. Shi X. Lee P. D. Stevens M. M. (2021). 3D Printed Silica-Gelatin Hybrid Scaffolds of Specific Channel Sizes Promote Collagen Type II, Sox9 and Aggrecan Production from Chondrocytes. Mater. Sci. Eng. C 123, 111964. 10.1016/j.msec.2021.111964 33812592

[B101] Neufurth M. Wang X. Schröder H. C. Feng Q. Diehl-Seifert B. Ziebart T. (2014). Engineering a Morphogenetically Active Hydrogel for Bioprinting of Bioartificial Tissue Derived from Human Osteoblast-like SaOS-2 Cells. Biomaterials 35, 8810–8819. 10.1016/j.biomaterials.2014.07.002 25047630

[B102] Nocera A. D. Comín R. Salvatierra N. A. Cid M. P. (2018). Development of 3D Printed Fibrillar Collagen Scaffold for Tissue Engineering. Biomed. Microdevices. 20, 26. 10.1007/s10544-018-0270-z 29484567

[B103] Noor N. Shapira A. Edri R. Gal I. Wertheim L. Dvir T. (2019). 3D Printing of Personalized Thick and Perfusable Cardiac Patches and Hearts. Adv. Sci. 6, 1900344. 10.1002/advs.201900344 PMC6548966 31179230

[B104] Nulty J. Freeman F. E. Browe D. C. Burdis R. Ahern D. P. Pitacco P. (2021). 3D Bioprinting of Prevascularised Implants for the Repair of Critically-Sized Bone Defects. Acta Biomater. 126, 154–169. 10.1016/j.actbio.2021.03.003 33705989

[B105] Ozbolat I. T. Hospodiuk M. (2016). Current Advances and Future Perspectives in Extrusion-Based Bioprinting. Biomaterials 76, 321–343. 10.1016/j.biomaterials.2015.10.076 26561931

[B106] Park J. Y. Choi J.-C. Shim J.-H. Lee J.-S. Park H. Kim S. W. (2014). A Comparative Study on Collagen Type I and Hyaluronic Acid Dependent Cell Behavior for Osteochondral Tissue Bioprinting. Biofabrication 6, 035004. 10.1088/1758-5082/6/3/035004 24758832

[B107] Park S. Shou W. Makatura L. Matusik W. Fu K. (2022). 3D Printing of Polymer Composites: Materials, Processes, and Applications. Matter 5, 43–76. 10.1016/j.matt.2021.10.018

[B108] Pati F. Jang J. Ha D.-H. Won Kim S. Rhie J.-W. Shim J.-H. (2014). Printing Three-Dimensional Tissue Analogues with Decellularized Extracellular Matrix Bioink. Nat. Commun. 5, 3935. 10.1038/ncomms4935 24887553 PMC4059935

[B109] Perez-Valle A. Del Amo C. Andia I. (2020). Overview of Current Advances in Extrusion Bioprinting for Skin Applications. Ijms 21, 6679. 10.3390/ijms21186679 PMC7555324 32932676

[B110] Petta D. Grijpma D. W. Alini M. Eglin D. D’Este M. (2018). Three-Dimensional Printing of a Tyramine Hyaluronan Derivative with Double Gelation Mechanism for Independent Tuning of Shear Thinning and Postprinting Curing. ACS Biomater. Sci. Eng. 4, 3088–3098. 10.1021/acsbiomaterials.8b00416 33435028

[B111] Piluso S. Skvortsov G. A. Altunbek M. Afghah F. Khani N. Koç B. (2021). 3D Bioprinting of Molecularly Engineered PEG-Based Hydrogels Utilizing Gelatin Fragments. Biofabrication 13, 045008. 10.1088/1758-5090/ac0ff0 34192670

[B112] Poldervaart M. T. Goversen B. De Ruijter M. Abbadessa A. Melchels F. P. W. Öner F. C. (2017). 3D Bioprinting of Methacrylated Hyaluronic Acid (MeHA) Hydrogel with Intrinsic Osteogenicity. PLoS One 12, e0177628. 10.1371/journal.pone.0177628 28586346 PMC5460858

[B113] Pourchet L. J. Thepot A. Albouy M. Courtial E. J. Boher A. Blum L. J. (2017). Human Skin 3D Bioprinting Using Scaffold-free Approach. Adv. Healthc. Mat. 6, 1601101. 10.1002/adhm.201601101 27976537

[B114] Ramakrishnan R. Kasoju N. Raju R. Geevarghese R. Gauthaman A. Bhatt A. (2022). Exploring the Potential of Alginate-Gelatin-Diethylaminoethyl Cellulose-Fibrinogen Based Bioink for 3D Bioprinting of Skin Tissue Constructs. Carbohydr. Polym. Technol. Appl. 3, 100184. 10.1016/j.carpta.2022.100184

[B115] Ratheesh G. Vaquette C. Xiao Y. (2020). Patient‐Specific Bone Particles Bioprinting for Bone Tissue Engineering. Adv. Healthc. Mat. 9, 2001323. 10.1002/adhm.202001323 33166078

[B116] Rauf S. Susapto H. H. Kahin K. Alshehri S. Abdelrahman S. Lam J. H. (2021). Self-assembling Tetrameric Peptides Allow *In Situ* 3D Bioprinting under Physiological Conditions. J. Mat. Chem. B 9, 1069–1081. 10.1039/d0tb02424d 33406193

[B117] Reinke J. M. Sorg H. (2012). Wound Repair and Regeneration. Eur. Surg. Res. 49, 35–43. 10.1159/000339613 22797712

[B118] Ricard-Blum S. (2011). The Collagen Family. Cold Spring Harb. Perspect. Biol. 3, a004978. 10.1101/cshperspect.a004978 21421911 PMC3003457

[B119] Robinson T. M. Talebian S. Foroughi J. Yue Z. Fay C. D. Wallace G. G. (2020). Fabrication of Aligned Biomimetic Gellan Gum-Chitosan Microstructures through 3D Printed Microfluidic Channels and Multiple *In Situ* Cross-Linking Mechanisms. ACS Biomater. Sci. Eng. 6, 3638–3648. 10.1021/acsbiomaterials.0c00260 33463177

[B120] Roh H.-H. Kim H.-S. Kim C. Lee K.-Y. (2021). 3D Printing of Polysaccharide-Based Self-Healing Hydrogel Reinforced with Alginate for Secondary Cross-Linking. Biomedicines 9, 1224. 10.3390/biomedicines9091224 34572410 PMC8471923

[B121] Romanazzo S. Molley T. G. Nemec S. Lin K. Sheikh R. Gooding J. J. (2021). Synthetic Bone‐Like Structures through Omnidirectional Ceramic Bioprinting in Cell Suspensions. Adv. Funct. Mat. 31, 2008216. 10.1002/adfm.202008216

[B122] Sánchez-Salazar M. G. Álvarez M. M. Trujillo-De Santiago G. (2021). Advances in 3D Bioprinting for the Biofabrication of Tumor Models. Bioprinting 21, e00120. 10.1016/j.bprint.2020.e00120

[B123] Schipani R. Scheurer S. Florentin R. Critchley S. E. Kelly D. J. (2020). Reinforcing Interpenetrating Network Hydrogels with 3D Printed Polymer Networks to Engineer Cartilage Mimetic Composites. Biofabrication 12, 035011. 10.1088/1758-5090/ab8708 32252045

[B124] Serpooshan V. Mahmoudi M. Hu D. A. Hu J. B. Wu S. M. (2017). Bioengineering Cardiac Constructs Using 3D Printing. J. 3D Print. Med. 1, 123–139. 10.2217/3dp-2016-0009

[B125] Shim J.-H. Jang K.-M. Hahn S. K. Park J. Y. Jung H. Oh K. (2016). Three-dimensional Bioprinting of Multilayered Constructs Containing Human Mesenchymal Stromal Cells for Osteochondral Tissue Regeneration in the Rabbit Knee Joint. Biofabrication 8, 014102. 10.1088/1758-5090/8/1/014102 26844597

[B126] Shin J. H. Kang H.-W. (2018). The Development of Gelatin-Based Bio-Ink for Use in 3D Hybrid Bioprinting. Int. J. Precis. Eng. Manuf. 19, 767–771. 10.1007/s12541-018-0092-1

[B127] Shin Y. J. Shafranek R. T. Tsui J. H. Walcott J. Nelson A. Kim D.-H. (2021). 3D Bioprinting of Mechanically Tuned Bioinks Derived from Cardiac Decellularized Extracellular Matrix. Acta Biomater. 119, 75–88. 10.1016/j.actbio.2020.11.006 33166713

[B128] Shoulders M. D. Raines R. T. (2009). Collagen Structure and Stability. Annu. Rev. Biochem. 78, 929–958. 10.1146/annurev.biochem.77.032207.120833 19344236 PMC2846778

[B129] Si H. Xing T. Ding Y. Zhang H. Yin R. Zhang W. (2019). 3D Bioprinting of the Sustained Drug Release Wound Dressing with Double-Crosslinked Hyaluronic-Acid-Based Hydrogels. Polymers 11, 1584. 10.3390/polym11101584 PMC6835267 31569810

[B130] Sitarski A. M. Fairfield H. Falank C. Reagan M. R. (2018). 3D Tissue Engineered *In Vitro* Models of Cancer in Bone. ACS Biomater. Sci. Eng. 4, 324–336. 10.1021/acsbiomaterials.7b00097 29756030 PMC5945209

[B131] Sorushanova A. Delgado L. M. Wu Z. Shologu N. Kshirsagar A. Raghunath R. (2019). The Collagen Suprafamily: From Biosynthesis to Advanced Biomaterial Development. Adv. Mat. 31, 1801651. 10.1002/adma.201801651 30126066

[B132] Sun K. Li R. Li H. Li D. Jiang W. (2018). Comparison of Three-Dimensional Printing for Fabricating Silk Fibroin-Blended Scaffolds. Int. J. Polym. Mater. Polym. Biomaterials 67, 480–486. 10.1080/00914037.2017.1354204

[B133] Sung K. Patel N. R. Ashammakhi N. Nguyen K. L. (2021). 3-Dimensional Bioprinting of Cardiovascular Tissues: Emerging Technology. JACC Basic Transl. Sci. 6, 467–482. 10.1016/j.jacbts.2020.12.006 34095635 PMC8165127

[B134] Unterman S. A. Gibson M. Lee J. H. Crist J. Chansakul T. Yang E. C. (2012). Hyaluronic Acid-Binding Scaffold for Articular Cartilage Repair. Tissue Eng. Part A 18, 2497–2506. 10.1089/ten.TEA.2011.0711 22724901 PMC3501122

[B135] Uz M. Donta M. Mededovic M. Sakaguchi D. S. Mallapragada S. K. (2019). Development of Gelatin and Graphene-Based Nerve Regeneration Conduits Using Three-Dimensional (3D) Printing Strategies for Electrical Transdifferentiation of Mesenchymal Stem Cells. Ind. Eng. Chem. Res. 58, 7421–7427. 10.1021/acs.iecr.8b05537

[B136] Wakitani S. Goto T. Pineda S. J. Young R. G. Mansour J. M. Caplan A. I. (1994). Mesenchymal Cell-Based Repair of Large, Full-Thickness Defects of Articular Cartilage. J. Bone & Jt. Surg. 76, 579–592. 10.2106/00004623-199404000-00013 8150826

[B137] Wang B. Liu S. Xie Y.-Y. (2019). Role and Prospects of Regenerative Biomaterials in the Repair of Spinal Cord Injury. Neural Regen. Res. 14, 1352–1363. 10.4103/1673-5374.253512 30964053 PMC6524500

[B138] Watt F. M. Huck W. T. S. (2013). Role of the Extracellular Matrix in Regulating Stem Cell Fate. Nat. Rev. Mo.l Cell. Biol. 14, 467–473. 10.1038/nrm3620 23839578

[B139] Wehrle M. Koch F. Zimmermann S. Koltay P. Zengerle R. Stark G. B. (2019). Examination of Hydrogels and Mesenchymal Stem Cell Sources for Bioprinting of Artificial Osteogenic Tissues. Cel. Mol. Bioeng. 12, 583–597. 10.1007/s12195-019-00588-x

[B140] Wiggenhauser P. S. Schwarz S. Koerber L. Hoffmann T. K. Rotter N. (2019). Addition of Decellularized Extracellular Matrix of Porcine Nasal Cartilage Improves Cartilage Regenerative Capacities of PCL-Based Scaffolds *In Vitro* . J. Mater Sci. Mater Med. 30, 121. 10.1007/s10856-019-6323-x 31655914

[B141] Won J.-Y. Lee M.-H. Kim M.-J. Min K.-H. Ahn G. Han J.-S. (2019). A Potential Dermal Substitute Using Decellularized Dermis Extracellular Matrix Derived Bio-Ink. Artif. Cells, Nanomedicine, Biotechnol. 47, 644–649. 10.1080/21691401.2019.1575842 30873886

[B142] Xing H. Lee H. Luo L. Kyriakides T. R. (2020). Extracellular Matrix-Derived Biomaterials in Engineering Cell Function. Biotechnol. Adv. 42, 107421. 10.1016/j.biotechadv.2019.107421 31381963 PMC6995418

[B143] Xu Y. Hu Y. Liu C. Yao H. Liu B. Mi S. (2018). A Novel Strategy for Creating Tissue-Engineered Biomimetic Blood Vessels Using 3D Bioprinting Technology. Materials 11, 1581. 10.3390/ma11091581 PMC6163305 30200455

[B144] Yan M. Lewis P. L. Shah R. N. (2018). Tailoring Nanostructure and Bioactivity of 3D-Printable Hydrogels with Self-Assemble Peptides Amphiphile (PA) for Promoting Bile Duct Formation. Biofabrication 10, 035010. 10.1088/1758-5090/aac902 29848794

[B145] Ye W. Li H. Yu K. Xie C. Wang P. Zheng Y. (2020). 3D Printing of Gelatin Methacrylate-Based Nerve Guidance Conduits with Multiple Channels. Mater. Des. 192, 108757. 10.1016/j.matdes.2020.108757

[B146] Yu C. Ma X. Zhu W. Wang P. Miller K. L. Stupin J. (2019). Scanningless and Continuous 3D Bioprinting of Human Tissues with Decellularized Extracellular Matrix. Biomaterials 194, 1–13. 10.1016/j.biomaterials.2018.12.009 30562651 PMC6339581

[B147] Yue B. (2014). Biology of the Extracellular Matrix. J. Glaucoma 23, S20–S23. 10.1097/IJG.0000000000000108 25275899 PMC4185430

[B148] Yun J. Lee J. Ha C. W. Park S. J. Kim S. Koo K. T. (2021). The Effect of 3‐D Printed Polylactic Acid Scaffold with and without Hyaluronic Acid on Bone Regeneration. J. Periodontol, 1–11. 10.1002/JPER.21-0428 34773704

[B149] Zhang J. Eyisoylu H. Qin X.-H. Rubert M. Müller R. (2021). 3D Bioprinting of Graphene Oxide-Incorporated Cell-Laden Bone Mimicking Scaffolds for Promoting Scaffold Fidelity, Osteogenic Differentiation and Mineralization. Acta Biomater. 121, 637–652. 10.1016/j.actbio.2020.12.026 33326888

[B150] Zhang X. Liu Y. Luo C. Zhai C. Li Z. Zhang Y. (2021). Crosslinker-free Silk/decellularized Extracellular Matrix Porous Bioink for 3D Bioprinting-Based Cartilage Tissue Engineering. Mater. Sci. Eng. C 118, 111388. 10.1016/j.msec.2020.111388 33254994

[B151] Zhao X. Lang Q. Yildirimer L. Lin Z. Y. Cui W. Annabi N. (2016). Photocrosslinkable Gelatin Hydrogel for Epidermal Tissue Engineering. Adv. Healthc. Mat. 5, 108–118. 10.1002/adhm.201500005 PMC4608855 25880725

[B152] Zhong C. Xie H.-Y. Zhou L. Xu X. Zheng S.-S. (2016). Human Hepatocytes Loaded in 3D Bioprinting Generate Mini-Liver. Hepatobiliary Pancreat. Dis. Int. 15, 512–518. 10.1016/s1499-3872(16)60119-4 27733321

[B153] Zhu K. Shin S. R. Van Kempen T. Li Y. C. Ponraj V. Nasajpour A. (2017). Gold Nanocomposite Bioink for Printing 3D Cardiac Constructs. Adv. Funct. Mat. 27, 1605352. 10.1002/adfm.201605352 PMC6181228 30319321

